# Metagenomic assembly reveals hosts and mobility of common antibiotic resistome in animal manure and commercial compost

**DOI:** 10.1186/s40793-022-00437-x

**Published:** 2022-08-11

**Authors:** Tianlei Qiu, Linhe Huo, Yajie Guo, Min Gao, Guoliang Wang, Dong Hu, Cheng Li, Zhanwu Wang, Guiming Liu, Xuming Wang

**Affiliations:** 1 Beijing Key Laboratory of Agricultural Genetic Resources and Biotechnology, Institute of Biotechnology, Beijing Academy of Agriculture and Forestry Sciences, Beijing, 100097 People’s Republic of China; 2 Institute of Agro-Resources and Environment, Hebei Fertilizer Technology Innovation Center, Hebei Academy of Agriculture and Forestry Sciences, Shijiazhuang, 050051 Hebei People’s Republic of China; 3 Institute of Quality Standard and Testing, Beijing Academy of Agriculture and Forestry Sciences, Beijing, 100097 People’s Republic of China

**Keywords:** Metagenome, Antibiotic resistance genes, Resistome, Host community, Mobile genetic elements, Livestock farm

## Abstract

**Background:**

Antibiotics and antibiotic resistance genes (ARGs) used in intensive animal farming threaten human health worldwide; however, the common resistome, ARG mobility, and ARG host composition in different animal manures and mixed manure composts remain unclear. In the present study, metagenomic assembly and cross-sample mapping were used to comprehensively decipher the common resistome and its potential mobility and hosts in animal manure and composts.

**Results:**

In total, 201 ARGs were shared among different animal (layer, broiler, swine, beef cow, and dairy cow) manures and accounted for 86–99% of total relative abundance of ARGs. Except for multidrug, sulfonamide, and trimethoprim resistance genes, the relative abundance of most ARGs in composts was significantly lower than that in animal manure. Procrustes analysis indicated that antibiotic residues positively correlated with ARG composition in manure but not in composts. More than 75% ARG subtypes were shared between plasmids and chromosomes in our samples. Transposases could play a pivotal role in mediating the transfer of ARGs between different phyla in animal manure and composting. Cross-sample mapping to contigs carrying ARGs showed that the hosts of common resistome in manure had preference on animal species, and the dominant genus of ARG host shifted from *Enterococcus* in manure to *Pseudomonas* in composts. The broad host range and linking with diverse mobile genetic elements (MGEs) were two key factors for ARGs, such as *sul1* and *aadA*, which could survive during composting. The multidrug resistance genes represented the dominant ARGs in pathogenic antibiotic-resistant bacteria in manure but could be effectively controlled by composting.

**Conclusions:**

Our experiments revealed the common resistome in animal manure, classified and relative quantified the ARG hosts, and assessed the mobility of ARGs. Composting can mitigate ARGs in animal manure by altering the bacterial hosts; however, persistent ARGs can escape from the removal because of diverse host range and MGEs. Our findings provide an overall background for source tracking, risk assessment, and control of livestock ARGs.

**Supplementary Information:**

The online version contains supplementary material available at 10.1186/s40793-022-00437-x.

## Background

Antibiotics have been widely used in livestock husbandry for growth promotion and prophylactic purposes or for treating animal diseases since they were initially approved as feed additives by the U.S. Food and Drug Administration in 1950. Approximately 162,000 tons of antibiotics are consumed by livestock and poultry annually in China, accounting for > 52% of the total antibiotic usage [1]. Most of these antibiotics are poorly metabolized by animals, resulting in abundant residues in feces leading to the development of antibiotic-resistant bacteria (ARB) and associated resistance genes (ARGs) [2, 3]. Increasing research reveals that livestock and poultry manure are reservoirs for ARB, ARG, and potential human pathogenic bacteria (HPB) dissemination. China is the largest poultry and pig producer worldwide [4], with approximately 3.8 billion tons of livestock and poultry manure production per year [5]. Manure aerobic composting is a commonly employed technology to decompose complex organic compounds into steady end-products that can be used as organic fertilizers for agricultural soil amendment. Previous studies have focused on ARG changes and potential hosts during composting with single animal manure [6, 7]. However, mixed manure instead of single manure is often used in commercial organic fertilizers [8]. Therefore, the investigation of the common antibiotic resistome in different animal manures and commercial composts could reveal important information for preventing ARG pollution and spread from farm animals.

ARG enrichment in the environment is widespread and mainly occurs through horizontal gene transfer (HGT) mediated by mobile genetic elements (MGEs), including plasmids, transposons, integrons, and integrative conjugative elements (ICEs) [9–11]. ARGs and MGEs in animal manure and composting processes have been well documented in terms of diversity and abundance [7, 12–14]; however, the common resistome and mobile ARGs of different manures and commercial composts remain to be elucidated, which hinders the comprehensive understanding of the manure resistome and associated risk.

The bacterial community is a key factor affecting ARG profiles during composting [14]. Clarifying ARG hosts is conducive to controlling ARG dissemination during manure composting. The identification and quantification of potential ARG host bacteria are often based on correlation analysis [7, 15–20]. However, because the 16S rDNA amplicon sequencing did not include ARG information, the quantification based on the co-occurrence of ARGs and bacteria could have overestimated the relative abundance of potential ARG hosts. The identification and quantitative analysis of contigs carrying ARGs (ARCs) could provide a feasible method for analysis of ARG hosts in the environment.

In this study, we used metagenomic assembly combined with relative quantitative analysis of ARCs to build ARG inventories in 29 farms and 13 commercial composting plants revealing the fate of ARG hosts in mixed manure composting. By comparing ARG relative abundance in manure and composts, we determined the common resistome of animal manure and its variation after composting. Through metagenomic assembly and co-localization analysis, we obtained ARGs and additional information on co-located genes to predict ARG mobility and virulence. Cross-sample mapping based on all no-redundant ARCs of all samples was used to quantify ARG hosts. The results revealed commercial composting strongly influence the common antibiotic resistome in farm animal manure. Our insights provide useful guidance for risk assessment and control of farm-sourced ARG spread at an industrial scale.

## Methods

A full version of the Methods is available in the Supplementary Information (SI). The metagenomic analysis is described in detail in the Additional file 1: Fig. S1 and SI.

### Sample collection

Manure and compost samples were collected from 29 concentrated animal feeding operations farms and 13 commercial composting plants from northern China between October 2017 and October 2018 (Additional file 1: Fig. S2 and Table S1).

### Determination of antibiotic concentration

The 19 antibiotics analyzed in this work belong to five categories and were mainly selected from those used for livestock and poultry animals [1]. Antibiotics were pretreated and analyzed according to Li et al.’s study [21].

### DNA extraction, metagenomic sequencing, and quality analysis

DNA was extracted using the MoBio PowerFecal DNA isolation kit (Mobio Laboratories, Carlsbad, CA, USA) according to the manufacturer’s protocol. The DNA extracts was used for shotgun sequencing using the Illumina Novaseq 6000. The raw data using Readfq V8 (https://github.com/cjfields/readfq) was preprocessed to acquire clean data for subsequent analyses. Sequencing data were deposited in the Genome Sequence Archive repository under BioProject number CRA005191.

### Calculating ARG abundance and statistics

ARG abundance was determined using ARGs-OAP v2.0, which integrates the detection of ARGs using the reference database SARG.2.2 [22]. ARGs were quantified by normalizing ARG abundance to the copy number of the 16S rRNA gene [23].

The Tukey HSD test, t-test or wilcox.test was conducted to analyze the statistical differences. The correlation matrix was constructed with ARG and antibiotics by calculating all pairwise Spearman’s rank correlations. The resulting correlation matrixes were translated into an association network using Gephi 0.9.2 [24]. The protest in the R vegan package was conducted to Procrustes between antibiotic residues and ARGs in all samples.

### Metagenomic data analysis

Kraken2 and Bracken was used to obtain the taxonomic profile of each sample based on filtered metagenomic data [25, 26]. Principal coordinate analysis (PCoA) was used to estimate the community dissimilarities.

Metagenomes were assembled using MEGAHIT [27]; thereafter, all contigs over 500 bp were retained and merged at 95% identity using CD-HIT-EST [28]. The N50 of the non-redundant contigs was 1723 bp and the L50 was 1 150 602 bp. For identifying antibiotic resistance contigs (ARCs), the DIAMOND was used to search the protein sequences of the SARG.2.2 database. For the multiple ARGs annotated, genes were predicted on the ARGs using Prokka [29]. ARG-like open reading frames (ORFs) on ARCs were determined against the SARG.2.2 database [30]. The occurrence of ARCs in chromosomes or plasmids was determined using BLASTn against NCBI Ref of 14,827 complete bacterial genome and 33,580 complete plasmid (until July 2021). The virulence genes were identified by comparing ARCs against VFdb [31].

For MGEs, one ORF was considered as a transposon or recombinase gene if one of the following keywords was in its best BLAST hit description: transposase, transposon, or recombinase [32]. Integrons in ARCs were identified using the IntegronFinder [33].

The abundance of non-redundant contigs was determined using Salmon [34]. The relative abundance of contigs was presented with contigs per kilobase per million mapped reads (*cpm*) according to the following equation:

$$cpm = 1{,}000{,}000*\frac{reads\; mapped\; to\; contig/contig\; length}{{sum\left( {reads\; mapped\; to\; contig/contig \;length} \right)}}$$

The relative abundance of ARCs was extracted from the whole quantification table of all non-redundant contigs. The ARCs were first taxonomically classified using Kraken2 in metaWARP [35].

The relative abundance of ARG hosts at the phylum or genus level were calculated by summing the *cpm* of ARCs under the specific taxonomic group. Gephi software was used to visualize the bipartite and ARG host networks.

## Results

### ARG profile in manure from different animals

**Fig. 1:**
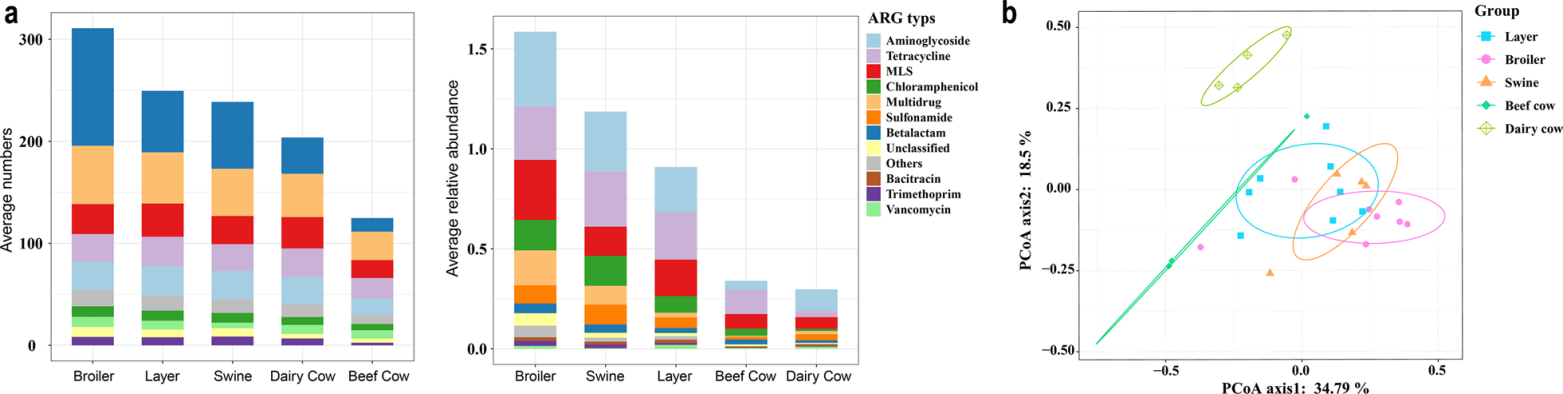
Antibiotic resistance gene (ARG) profiles in manures from different animals. **a** The average detected numbers and relative abundance (normalized by 16S rDNA) of ARG subtypes. **b** Principal co-ordinates analysis (PCoA) depicting the overall pattern of ARGs

In manure samples from 29 farms, 20 types of ARGs comprising 626 subtypes were detected; 545 in broiler manure, 427 in swine manure, 419 in layer manure, 288 in dairy cow manure, and 232 in cattle manure. Moreover, 86.58% of the ARGs belonged to the top five types, i.e., beta-lactam, multidrug, macrolide, aminoglycoside, and tetracycline resistance genes; these types accounted for 74.29% of the total ARG abundance (Fig. 1a). The sulfonamide resistance genes comprised three subtypes; however, they accounted for 6.68% of the total ARG abundance. In contrast, the beta-lactam resistance genes accounted for 55.11% of the total ARGs, but they only accounted for 3.47% of the total ARG abundance. The average number (311) and relative abundance (1.59 per 16S rRNA gene) of total ARGs in broiler manure revealed the highest values in manure samples.

The PCoA-based relative abundances of the ARG subtypes revealed that the profile of ARGs in broiler manure was more similar to that in swine manure (Fig. 1b). As the dairy cow manure had the lowest antibiotic residue in all samples (Additional file 2: Dataset 1), the profiles of ARGs in dairy cow manure differed from those in manure from other animals.

### Manure common resistome

**Fig. 2:**
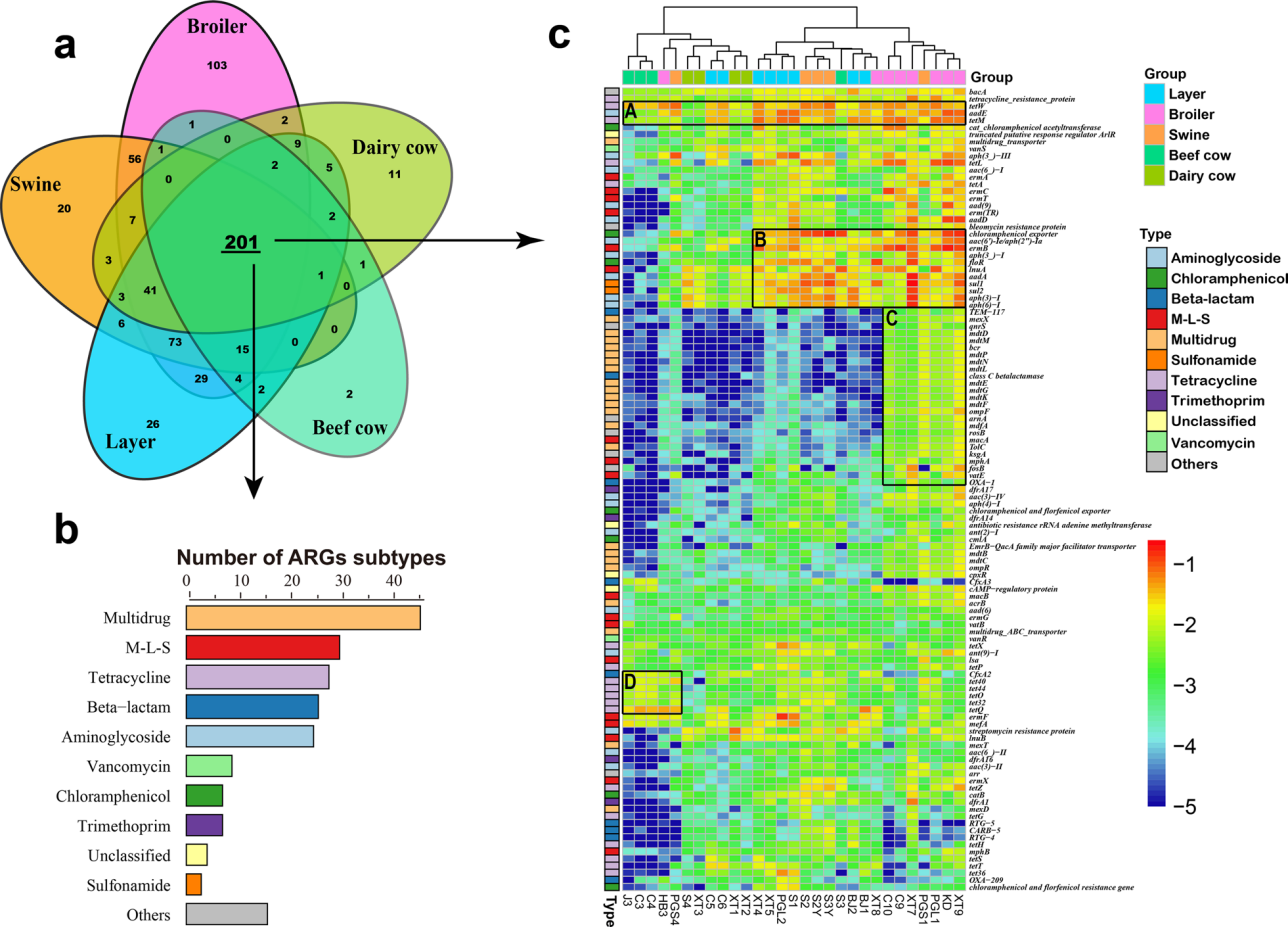
Venn diagram and heatmap of ARG subtypes in manure common resistome. **a** Venn diagram depicts the numbers of shared ARG subtypes. **b** The composition of common resistome (201 ARG subtypes); **c** The heatmap of common resistome ARGs (113 of 201 ARG subtypes), wherein average relative abundance was greater than 0.001 copies per 16S rRNA gene. (A) dominant ARG subtypes in all samples; (B) dominant ARG subtypes in manure samples except cow manure; (C) dominant ARG subtypes in broiler manure; (D) dominant ARG subtypes in beef cow manure

The 201 subtypes of ARGs, mainly comprising multidrug (46), M-L-S (30), tetracycline (28), beta-lactam (26), and aminoglycoside (25) resistance genes, were shared among different animal manures (Fig. 2). Moreover, these subtypes accounted for the majority of the total ARG relative abundance in manure samples; layer (98–99%), broiler (89–99%), swine (86–99%), and beef and dairy cow (> 99%). Here, we defined these 201 subtype ARGs as the common resistome in animal manure. The chloramphenicol exporter genes exhibited the highest average relative abundances (0.046 copies per 16S rRNA gene) in all samples, whereas the highest relative abundance (0.25 copies per 16S rRNA gene) of ARG type was found in sulfonamide resistance genes *sul1* in a broiler manure sample (Fig. 2c). Furthermore, genes such as *tetW* (0.0017–0.085 copies per 16S rRNA gene) and *tetM* (0.001–0.082 copies per 16S rRNA gene) that encode the ribosomal protection proteins and the aminoglycoside inactivation gene (*aadE*, 0.0022–0.093 copies per 16S rRNA gene) in all manure samples (Fig. 2c group A) were the most widely presented ARG subtypes. Aminoglycoside nucleotidyltransferase gene (*aadA*), streptomycin phosphotransferase genes (*AAC(6’)-Ie/APH(2’)-Ia*, *aph(3_) − I*, *aph(3) − I*, and *aph(6) − I*), and the M-L-S resistance genes (*ermB* and *lnuA*) were dominant in swine and chicken manure samples (group B). The broiler samples exhibited markedly higher relative abundances of multidrug efflux pump genes, such as *mdfA*, *mdtD*, *mdtE*, *mdtF mdtG*, *mdtK*, *mdtL*, *mdtM*, *mdtN*, *mdtP*, and *mexX* (group C) than the other samples. In contrast, some tetracycline resistance genes (*tetO*, *tetQ*, *tet32*, and *tet40*) and beta-lactamase gene (*cfxA2*) were specific to the beef cow manure (group D).

### Changes in the manure common resistome after composting

**Fig. 3:**
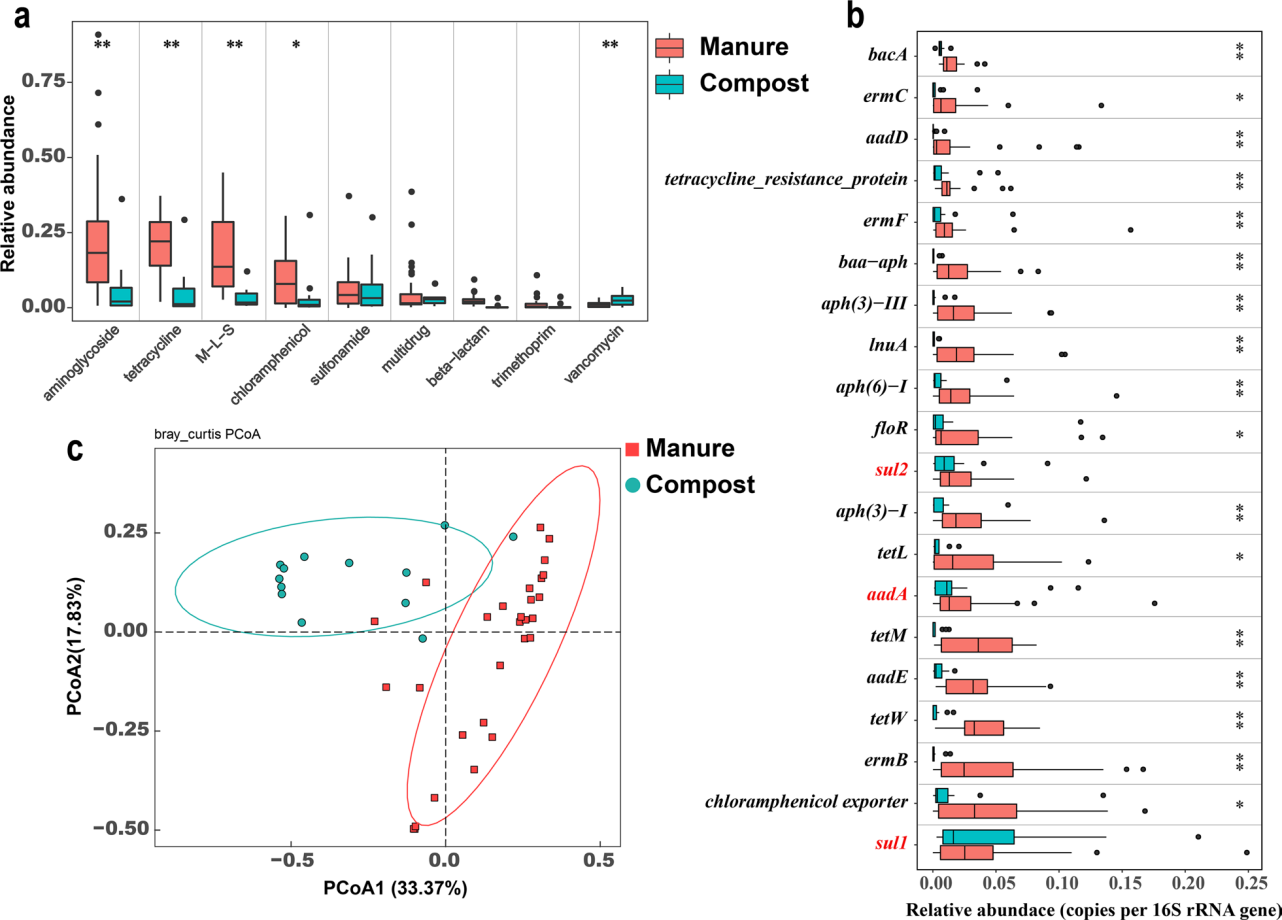
Manure common resistome shifted after composting. **a** Box-plot of ARG types between manure and compost. **b** Average relative abundances of the top 20 ARG subtypes in manure and compost. The asterisk “*”and “**” denote the significance level of 0.05 and 0.01, respectively. **c** Principal co-ordinates analysis (PCoA) depicts the shift pattern of ARG subtypes between manure and compost

Composting reduced the relative abundance of the main ARGs in the manure common resistome; the total relative abundance (0.938 copies per 16S rRNA gene) of ARGs in manure was significantly higher (*p* = 0.0016) than that (0.405 copies per 16S rRNA gene) in the composts (Additional file 1: Table S2). The relative abundances of 10/19 ARG types in composts were significantly lower than those in the manure samples; however, those of six other ARG types were significantly higher in composts than in the manure, but they all exhibited low relative abundances (Additional file 1: Table S2). Moreover, composting did not have any significant effect on three types (multidrug, sulfonamide, and trimethoprim resistance genes). The relative abundances of most main ARG types, such as aminoglycoside, chloramphenicol, M-L-S, tetracycline, and beta-lactam resistance genes, were significantly lower in composts than in manure (Fig. 3a), whereas that of vancomycin resistance genes was significantly higher in composts.

Furthermore, among the 201 common resistome ARGs, the relative abundances of 123 ARG subtypes were significantly lower in composts, whereas those of other 32 ARGs were significantly higher in composts than in manure. The relative abundances of the top 20 ARG subtypes were significantly lower in composts than in manure, except for two sulfonamide (*sul1* and *sul2*) and one aminoglycoside (*aadA*) resistance genes (Fig. 3b). Among all the ARG subtypes with average relative abundance > 0.001, only eight ARGs, including *vanR*, mutigrug_ABC_transporter, and *fosB*, had significantly higher relative abundance in composts than in manure (Additional file 1: Fig. S4). PCoA revealed that the composition of ARGs in most composts differed from that in the manure samples (Fig. 3c).

### Changes in the relationship between antibiotic residue and ARGs after composting

Nineteen antibiotics were detected in the manure samples, and they were classified into five categories, i.e., tetracyclines, quinolones, macrolides, sulfonamides, amphenicols, and two others (lincomycin and ceftiofur) (Additional file 2: Dataset 1). The average total concentrations of antibiotic residues in manures were 96,937 μg/kg (broiler), 50,248 μg/kg (swine), 5321 μg/kg (layer), 858 μg/kg (beef cow), and 295 μg/kg (dairy cow). Tetracyclines were the dominant antibiotics in swine and broiler manure, and they contributed 96.28% and 93.77% of the total concentration of antibiotics, respectively. Moreover, tetracyclines and quinolones accounted for 55.99% and 24.3% of the total concentrations in the layer manure. Quinolones were the dominant antibiotics in beef cow manure, contributing 89.77% of the total concentration of antibiotics.

**Fig. 4:**
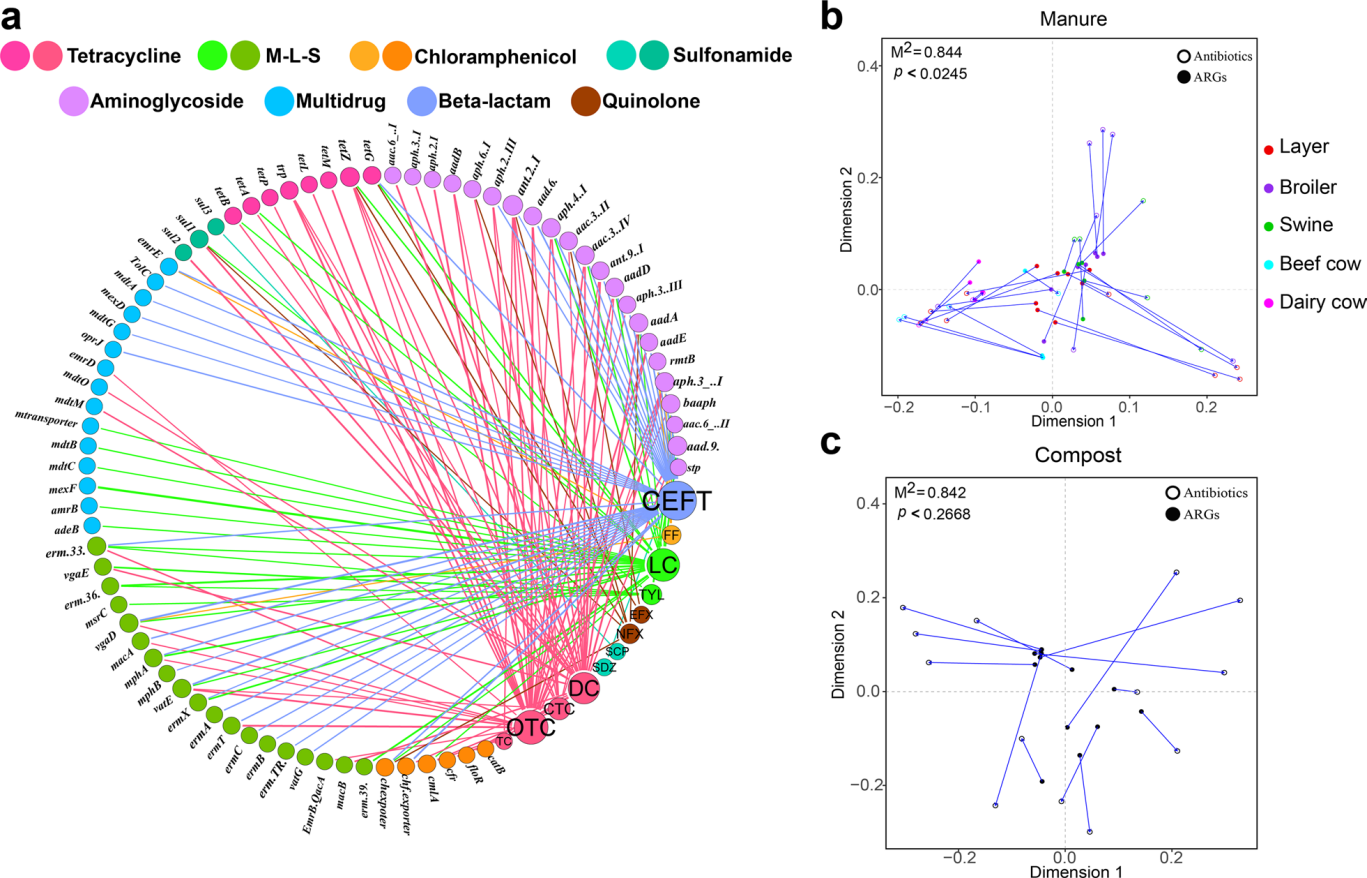
ARGs and antibiotic correlation analysis. **a** Network analysis based on the co-occurrence of ARGs and antibiotics (*r* > 0.5, *p* < 0.05). **b** Procrustes analysis indicating correlations between subtype ARG and antibiotics in manure. **c** Procrustes analysis indicating correlations between subtype ARG and antibiotics in compost. (sulfadiazine (SDZ), tetracycline (TC), chlortetracycline (CTC), oxytetracycline (OTC), doxycycline (DC), tylosin (TYL), lincomycin (LC), norfloxacin (NFX), enrofloxacin (EFX), florfenicol (FF), ceftiofur (CEFT))

We evaluated the correlations between ARGs and antibiotics in the manure samples. Significant positive correlations (*p* < 0.05, r > 0.5) were observed between the 12 antibiotics and six ARGs (Fig. 4a). The network revealed that most antibiotic residues were related to multiple ARG types. The dominant antibiotics (tetracyclines) in manure exhibited a significant positive correlation with all six ARG types, indicating that numerous antibiotic residues will directly lead to the development of antibiotic resistance in animal manure. Some low concentrations of antibiotics were also correlated with several ARGs. Ceftiofur is a therapeutic antibiotic, which has a low concentration in manure (0–561.42 μg/kg); however, it revealed a significant correlation with 37 ARG subtypes. Procrustes analysis confirmed that manure resistomes were significantly correlated with antibiotic residues (Fig. 4b); however, they were not correlated with antibiotics after composting (Fig. 4c). These results indicate that composting can reduce the antibiotic residues and decrease the correlation between antibiotics and resistance genes in composts.

### Changes in ARG bacterial hosts

**Fig. 5:**
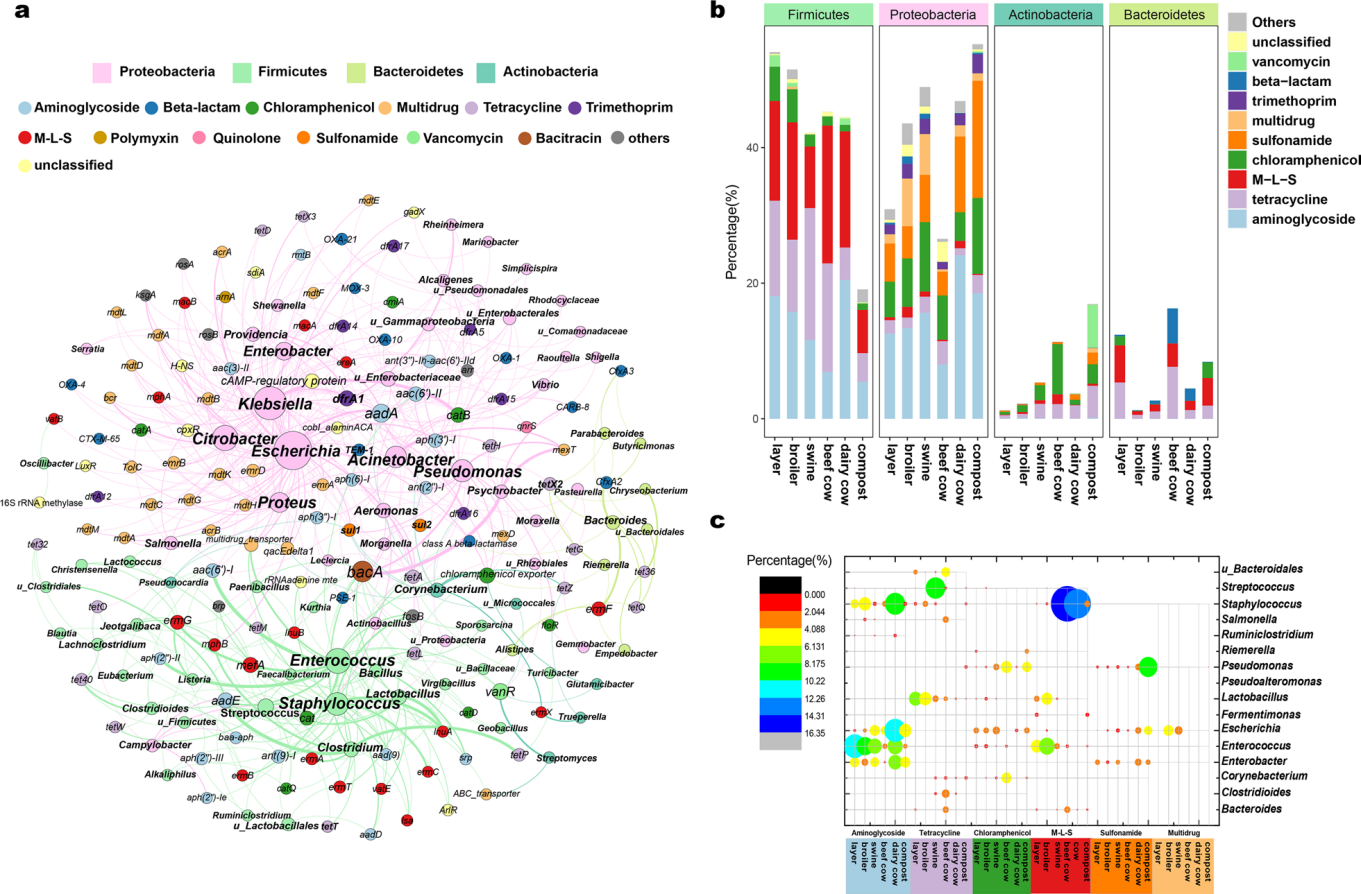
Host distribution of ARGs based on contig assembling and quantifying. **a** Network analysis based on the physical linkage pattern of ARGs and hosts on the antibiotic resistance contigs (ARCs). The nodes (ARGs or genus) which had at least two edges were retained. The nodes were colored according to ARG type and host phylum. **b** The distribution of ARG types in bacteria at the phylum level based on the cross-sample mapping to ARCs. **c** The major hosts (top 10 genera with average percentage in each sample) for M-L-S, tetracycline, aminoglycoside, chloramphenicol, and sulfonamide resistance genes

In total, 1200 non-redundant ARCs were assembled from all metagenomes, including 224 ARG subtypes. *Proteobacteria* and *Firmicutes* were the dominant hosts, accounting for 50.08% and 37.77% of the ARCs, respectively, whereas Bacteroidetes and Actinobacteria accounted for 6.49% and 5.24%, respectively (Additional file 1: Table S3). The host bacteria included pathogens, such as *Escherichia*, *Enterococcus*, *Klebsiella*, *Staphylococcus*, *Acinetobacter*, *Pseudomonas*, *Clostridium*, *Citrobacter*, *Streptococcus*, *and Enterobacter* (Fig. 5a). *Enterococcus* and *Escherichia* were the dominant genus, accounting for 10.4% and 10.23% of the total 1200 ARCs, respectively, whereas *Enterococcus* and *Escherichia* carried 17.4% and 33.04% of all detected 224 ARG subtypes, respectively (Additional file 1: Table S4). *Escherichia* carried the most diverse ARGs and it exhibited 74 ARG subtypes classified into 15 types of ARGs (Additional file 1: Table S4). Although the relative abundance (*cpm*) of all the host ARCs in manure decreased after composting, the hosts of the top list ARGs in manure and compost differed (Additional file 1: Fig. S5). In the composts, *Pseudomonas*, not *Enterococcus*, was the most abundant host; *Microbacterium* and *Riemerella* were included in the top 10 list in compost but not *Lactobacillus* and *Streptococcus* (Additional file 1: Fig. S5).

Moreover, based on the *cpm* of ARCs at genus level, the major hosts of the main ARG types, such as aminoglycoside, chloramphenicol, M-L-S, tetracycline, multidrug, and sulfonamide resistance genes were investigated (Additional file 1: Fig. S6). The major hosts of aminoglycoside resistance genes were *Enterococcus*, *Streptococcus*, and *Enterobacter*, whereas those of tetracycline resistance genes were *Pseudomonas*, *Lactobacillus*, and *Streptococcus*. Compared to other samples, the *cpm* of hosts for the six major types of ARGs in broiler and swine manure were significantly higher. *Escherichia* was the dominant host for multidrug resistance genes. The richness of ARG hosts at the genus level in compost was significantly lower than that in manure samples (Additional file 1: Fig. S7), while the Shannon and Simpson indices of ARG hosts did not exhibit a significant difference between manure and composts.

To observe the changes in the composition of the ARG hosts in manure after composting, the percentage of hosts was calculated based on the relative abundance (*cpm*) (Fig. 5b). According to the results of taxon classification on ARCs, we found that the hosts could change with different ARG types at the phylum level. The aminoglycoside resistance genes were mainly distributed in *Firmicutes* and *Proteobacteria*; however, the two phyla carrying aminoglycoside resistance genes were affected by composting; the average percentage of *Firmicutes* and *Proteobacteria* with aminoglycoside resistance genes decreased from 14.58 to 5.48% and increased from 14.73 to 18.55% after composting, respectively. Tetracycline resistance genes were widely distributed in all four phyla. Additionally, the percentage of the other three phylum hosts with tetracycline resistance genes decreased after composting; however, those of *Actinobacteria* carrying tetracycline resistance genes did not. *Firmicutes* mainly carried the M-L-S resistance genes, whereas *Proteobacteria* carried the sulfonamide, multidrug, and trimethoprim resistance genes. Notably, the percentage of *Proteobacteria* carrying sulfonamide resistance genes increased from 6.41% in manure to 17.28% in compost.

Based on the percentage of ARCs at the genus level, the dominant ARGs in animal manure and compost are illustrated in Fig. 5c. The percentage of *Enterococcus* carrying aminoglycoside resistance genes accounted for more than 6% in layer, broiler, swine, and dairy cow manure; however, after composting, this percentage decreased to 2.27%. *Staphylococcus* was the dominant ARG host in beef cow and dairy cow manure; it carried aminoglycoside resistance genes accounting for 10.18% of all hosts in dairy cow manure and M-L-S resistance genes accounting for 16.32% and 13.47% in beef cow manure and dairy cow manure, respectively. *Pseudomonas* carried sulfonamide resistance genes, accounting for 9.40% of the total ARG hosts in compost. Moreover, *Streptococcus* with tetracycline resistance genes was dominant, accounting for 9.79% of all hosts in swine manure. Furthermore, the dominant tetracycline resistance genes hosts were *Lactobacillus* in layer (6.49%) and broiler (6.11%) manure, thereby exhibiting high concentrations of tetracycline residue, similar to that in swine manure. The results indicated that veterinary antibiotics could cause antibiotic resistance in animal guts; however, the ARGs in different animal guts may have their own dominant hosts.

### Distribution of ARGs in mobile gene elements

**Fig. 6:**
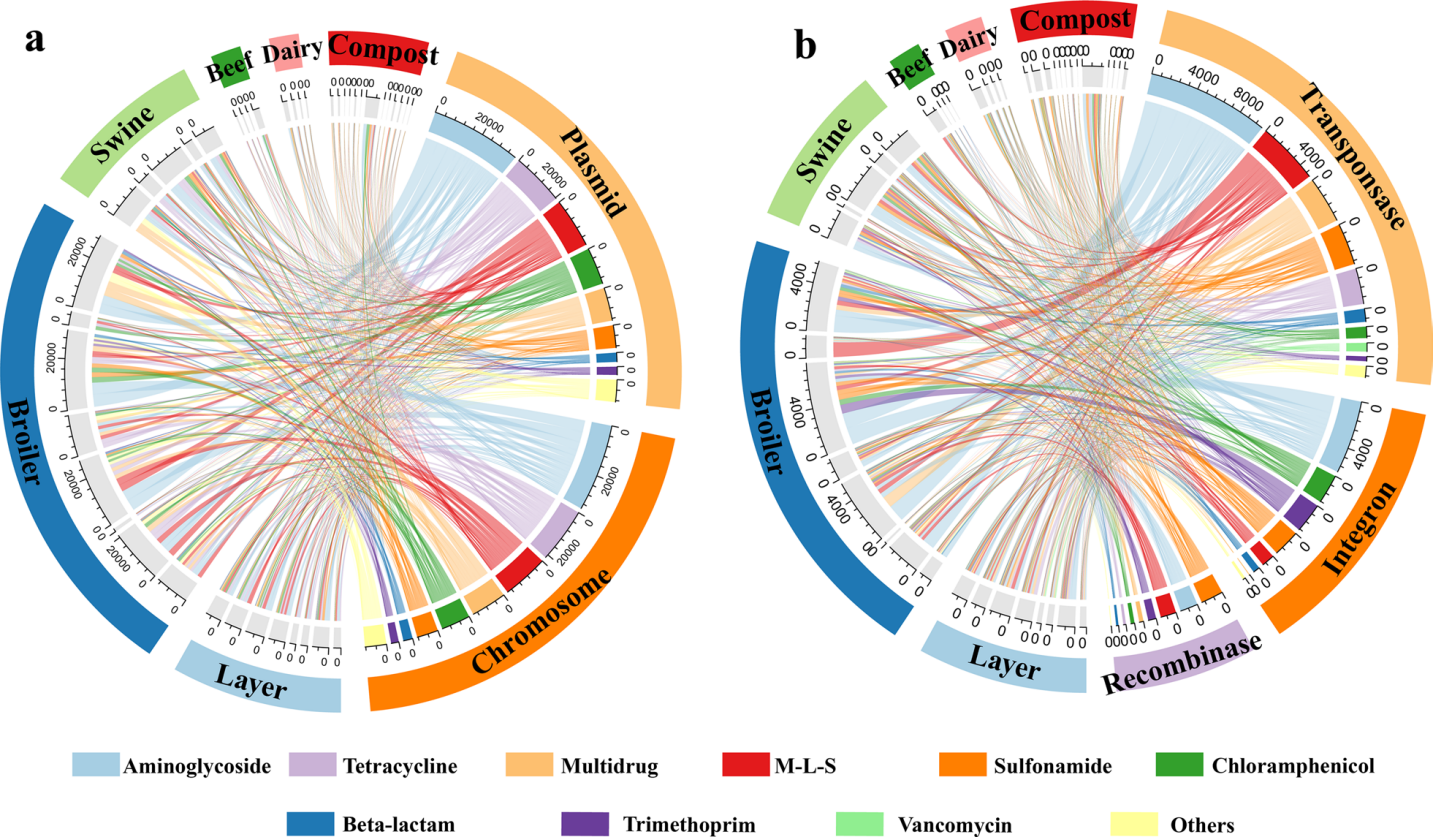
Circos representation showing the distribution of ARGs. **a** Distribution of ARGs harbored on plasmids and chromosomes; **b** distribution of ARGs linked with transposase, integron, and recombinase

In general, 695 (57.92%) of 1200 ARCs carried MEGs, including plasmids (43.68%), transposase (16.33%), integron (8.50%), and recombinase (3.08%); the detailed co-occurrence patterns of ARGs and MGEs are summarized in Additional file 1: Table S6. Moreover, 741 ARCs were located on plasmids or chromosomes, carrying 204 ARG subtypes classified into 19 ARG types. In our samples, 75.98% matched ARGs (155 subtypes) were shared between plasmids and chromosome, and all the top 20 high relative abundance ARG subtypes (Fig. 3b) were all shared by plasmids and chromosome (Additional file 1: Fig. S8). The 13 ARG subtypes comprising aminoglycoside, beta-lactam, tetracycline, M-L-S trimethoprim and polymyxin resistance genes were only found in the plasmids, whereas 36 ARG subtypes belonged to 11 ARG types including beta-lactam, multidrug, tetracycline, vancomycin and M-L-S resistance genes, were only found on the chromosomes. The circos diagram revealed that the plasmids had a similar relative abundance (*cpm*) of ARCs than the chromosomes (Fig. 6a). Aminoglycoside and tetracycline resistance genes were dominant in both plasmids and chromosomes. The chromosomes possessed more multidrug resistance genes, whereas plasmids carried more chloramphenicol and M-L-S resistance genes.

The heatmap reveals the changes in the relative abundance (*cpm*) of plasmid-associated or chromosome-associated ARCs in manure and compost (Additional file 1: Fig. S9). The multiple ARGs carrying contigs were almost belonged to the shared ARCs between plasmid and chromosome, and they are also persistent and dominant in composts, including tetracycline (*tetL* and *tetM*), sulfonamide (*sul1* and *sul2*), chloramphenicol (chloramphenicol exporter), and aminoglycoside (*aadA*, *aad(9)*, *aadE*, *aph(3)*, and *aph(6)*) resistance genes (Additional file 1: Fig. S9 group A). The multidrug resistance genes in shared ARCs (Additional file 1: Fig. S9 group B), which had a high relative abundance in meat animal (broiler and swine) manure, could be efficiently reduced by composting.

Of the 107 ARG subtypes, 85 subtypes were linked with transposase, 34 with integron, and 22 with recombinase (Additional file 1: Fig. S10). *Sul1* and *aadA*, which had high relative abundance in all manure samples and did not significantly reduce after composting, were linked with all three MGEs in our samples. This result indicated that compared with “single” ARGs, the ARGs connected with MGEs could exhibit a higher chance of survival during composting. The heatmaps based on relative abundance (*cpm*) revealed that integron-carried ARCs exhibited more multiple ARGs (30/100) than the transposase-carried (21/182) and recombinase-carried ARCs (4/37); furthermore, multiple ARGs revealed the highest relative abundance of integron-carried ARCs in composts (Additional file 1: Figs. S13–S15). Aminoglycoside resistance genes were dominant in all three MGEs carrying ARGs (Fig. 6b). The distribution of ARGs connected to the three MGEs was distinct. Transposase was associated with the highest number and relative abundance of ARGs in all three MGEs (Additional file 1: Fig. S10 and S13); moreover, the MLS-resistance genes, multidrug resistance genes, and tetracycline resistance genes were more easily found in transposase-related ARCs. The chloramphenicol resistance genes and trimethoprim resistance genes were two dominant ARGs in the integron-related ARCs. Among all the recombinase-carrying ARGs, sulfonamide resistance genes had the highest relative abundance. The taxon circos diagram depicts that the potential hosts of transposase-related ARCs were included in all four phyla, and 98.9% of the integron-carrying ARCs belonged to Proteobacteria (Additional file 1: Fig. S11).

## Discussion

Animal manure is an important source of ARGs in the environment, and 70% of the antibiotics are used in animals worldwide [36]. Direct use of raw manure on soil could significantly increase the abundance of ARGs [37]. Composting is a manure management practice, and its high temperature can effectively reduce the antibiotic residue in feces [38]; however, as ARGs could be duplicated or spread through HGTs, composting did not reveal consistent results with respect to reducing ARGs [39]. During composting, ARG maintenance is associated with HGT and shifts in related bacterial communities [18]. Thus, it is important to analyze the common resistome in animal manure and the changes in ARG-related hosts and MGEs after composting. In the present study, we investigated different livestock manures and their composts through metagenomic analysis to assess the composition of the common resistome and its potential hosts in manure, and to evaluate the potential hosts and mobility in the finished composts.

### Comparison of ARGs in animal manure and other environments

In general, 626 ARG subtypes were detected in our samples via metagenomic analysis, compared to the 109 ARG subtypes in different animal manure detected via high-throughput qPCR in a previous study [3]. The manure common resistome was found in our samples as well as in other niches, such as urban sewage, human feces, and farm soil [40–42]. Aminoglycoside and tetracycline resistance genes are abundant in human feces and animal manure [41]. Compared to urban sewage and human feces, animal manure had more multidrug resistance genes, accounting for 20.3% of the total ARGs, and a lower relative abundance of beta-lactam resistance genes (3.47%) [40]. The intensification of livestock operations caused a rapid increase in ARGs in chickens and pigs. In low- and middle-income countries, from 2000 to 2018, the percentage of multidrug-resistant bacteria increased from 0.15 to 0.41 in chicken and from 0.13 to 0.34 in pigs [43]. This indicates that the profiles of ARGs are often associated with selective pressure and spatiotemporal conditions [43]. PCoA-based relative abundance of the total bacteria and ARG hosts revealed that the profile of total bacteria in layer and broiler manure are similar; however, the profile of ARG hosts in broiler manure was closer to that in swine manure (Additional file 1: Fig. S12). Owing to the use of antimicrobial growth promoters in livestock, the long-term selection pressure of antibiotics has resulted in the emergence of multidrug resistance bacteria; moreover, multidrug resistance genes are more abundant in animal guts than in human guts, sewage, and soils.

### ARG host community determines ARG composition

The bacterial community can be controlled to limit the spread of ARGs by determining ARG host information. Moreover, ARGs could be directly linked to more specific bacterial hosts based on the assembled contigs or binning bacterial genomes via metagenomic analysis than by correlation analysis [44]. The correlation analysis majorly revealed that the main host phylum in composts was Actinobacteria [15, 44, 45]; however, in our assembled contigs, the dominant ARG host phylum was Proteobacteria (55.24%). Consistent with another study results [46], *Actinobacteria* also exhibited dominant relative abundance (51.71%) in our composts (Additional file 3: Dataset 2); however, the relative abundance of Actinobacteria hosts only accounted for 16.91% of all ARG hosts. This phenomenon indicated that most of the increased Actinobacteria did not carry ARGs after composting.

The antimicrobial growth promoters could exert pressure for selecting multidrug-resistant *E. coli* in the broiler guts [47]; therefore, in our samples, *E. coli* carried the most diverse ARGs and it was the dominant host of multidrug resistance genes in broiler and swine manure (Additional file 1: Table S4 and Fig. 5c). Moreover, it was related to diverse emerging human ARGs in various agricultural systems, such as chicken farms [41, 48, 49], swine farms [50, 51], dairy cow manure [51], and farming soils [52]. Another important ARG host in manure is *Enterococcus*, which is a major host of aminoglycoside resistance genes and M-L-S resistance genes in broiler and swine manure. Consistent with Xiong et al.’s research, *Enterococcus* was linked with vancomycin resistance genes (*vanR*, *vanS*) [49]. After composting, the relative abundance of ARG-carrying *Enterococcus* decreased from 832 to 16 cpm; however, *Enterococcus* exhibited a high abundance in the packaging area of a commercial composting plant; therefore, a suitable method should be followed for protecting workers from these potential ARG carrying pathogen [53]. Although *Lactobacillus* are commonly used as probiotics and are considered to be beneficial in the gut microbiota of animals, the tetracycline resistance and M-L-S resistance gene hosts were dominant in our broiler manure samples, which is consistent with the findings of studies on antibiotic susceptibility in *Lactobacillus* strains from poultry [54, 55]. Consistent with Keenum et al.’s results, *Staphylococcus* and *Streptococcus* were the major hosts for M-L-S and aminoglycoside resistance genes in beef and dairy cow manure [12]. Noteworthy, the relative abundance of *vanR* significantly increased after composting (Additional file 1: Fig. S3), because *vanR* was linked with several *Actinobacteria*, including *Streptomyces*, *Glutamicibacter*, u_*Micrococcales*, and some heat-tolerant *Firmicutes* genus such as *Bacillus* (Fig. 5a).

The constant or increased abundance of sulfonamide or trimethoprim resistance genes are often found during composting with various materials, such as swine manure [6, 7, 56], chicken manure [57, 58], cow manure [12, 16], sewage sludge [15], and food waste [18]. Previous correlation analysis showed that the increase in sulfonamide resistance genes was positively correlated with strains that were privileged during the cooling-maturation stages [7, 18], and the sulfonamide resistance genes were also often found to be correlated with MGEs, such as *intI1* or *ISR1* [7, 16, 57]. These results indicate that ARGs may be maintained in other strains due to HGTs. In our samples, based on the metagenomic analysis, we found two points that could be the reason for sulfonamide resistance genes to escape composting removal. 1. Sulfonamide resistance genes had diverse hosts (wide host range); *sul1* was carried by five genera belonging to *Proteobacteria*, *Firmicutes*, and *Actinobacteria* (Fig. 5a), including *Escherichia*, *Pseudomonas*, *Micrococcales*, *Pseudonocardia*, *Citrobacter*, *and Corynebacterium*. The quantification of contigs showed that the *Pseudonocardia*-like contig (XT2k141_483370) increased in the composts. *Pseudonocardia thermophiles* are thermophilic actinomycetes that can produce spores to resist high temperatures [59]. The major host analysis showed that *Pseudomonas*, instead of *Enterobacter* and *Escherichia*, became the dominant hosts of sulfonamide resistance genes after composting. The metatranscriptomic sequence and culture-dependent analysis both found that *Pseudomonas* was stable throughout the whole process of composting [12, 46, 60]. These results indicate that *sul1* could likely be maintained in thermotolerant bacteria during composting. 2. Sulfonamide resistance genes were co-located with all types of MGEs, including plasmids, integrons, transposons, and recombinase (Additional file 1: Figs. S8 and S10). The *cpm* of the dominant *sul1*-carrying contig (J5k141_381193) was located on the *Pseudomonas aeruginosa* plasmid and harbored *intI1* and IS6100 (Additional file 1: Fig. S16). The shared MGEs ensure that *sul1* has the potential to spread in multiple hosts by HGTs. Another ARG subtype, which may be maintained through HGTs in composts, is the aminoglycoside nucleotidyltransferase gene (*aadA*). In our samples, *aadA* was also linked with all MGEs (Additional file 1: Fig. S10), and its hosts were distributed across 20 genera belonging to the phyla *Proteobacteria* and *Actinobacteria* (Additional file 1: Table S5).

### Fate of potential pathogenic antibiotic-resistant bacteria

In total, 22 assembled ARCs were identified as VG-carrying contigs, which could be potential pathogenic antibiotic-resistant bacteria [61], and most of them belonged to *Enterobacteriaceae*, including *Citrobacter*, *Escherichia*, *Enterobacter*, and *Klebsiella*, except for *Enterococcus* (Additional file 1: Fig. S17; Additional file 4: Dataset 4). *Enterobacter* were also found as ARG- and VG-carried pathogens in long-term manure-amended greenhouse soils [62]. The ESKAPE pathogens (*Enterococcus faecium*, *Staphylococcus aureus*, *Klebsiella pneumoniae*, *Acinetobacter baumannii*, *Pseudomonas aeruginosa*, and *Enterobacter* species) are responsible for the majority of nosocomial infections globally and can acquire antibiotic resistance [63]. Although we found ARCs with virulence genes in *Enterococcus faecium*, *Klebsiella pneumoniae*, and *Enterobacter*, it is worth noting that we did not find any thermotolerant human pathogens such as *Staphylococcus aureus* and *Pseudomonas aeruginosa* relative ARCs with virulence genes in our composts. Most ARGs carried by potential pathogenic antibiotic-resistant bacteria were multidrug resistance genes, including *mdfA*, *mdtG*, *acrA*, and *acrB*. *emrD*, *emrE*, *emrB*-*qacA*, and *TolC* belong to the antibiotic efflux mechanism [64]. In contrast to Korin’s results [65] that virulence genes were associated with MGEs during food waste feeding and composting on poultry farms, most potential pathogenic antibiotic-resistant bacteria did not harbor MGEs, except for two contigs (*Enterococcus* and *Klebsiella*) linked with transposase genes and two contigs located on plasmids, so the potential pathogenic antibiotic-resistant bacteria in our manure samples may not have wide HGT possibility. Most potential pathogenic antibiotic-resistant bacteria were distributed in the high antibiotic residue manure, such as broilers, layers, and swine. Compared to that (31.13 cpm) in the manure samples, the average relative abundance (0.06 cpm) of potential pathogenic antibiotic-resistant bacteria in composts decreased significantly. Thus, composting has a good effect on controlling potential pathogenic antibiotic-resistant bacteria in manure.

### Gene mobility potentials of common antibiotic resistomes in manure and compost

HGT is an essential mechanism for the dissemination of ARGs in the environment via MGEs, such as plasmids, integrons, and transposons [11, 66, 67]. The current correlation analysis revealed that MGEs are important factors for eliminating ARGs during composting [14, 18, 20, 68]; however, there is a lack of direct linkage and evidence for the co-occurrence of ARGs and MGEs. In our samples, the top 20 high relative abundance ARGs were all harbored on plasmid and chromosome shared contigs (Additional file 1: Fig. S8) and most of persistent ARGs on composts were carried by shared ARCs (Additional file 1: Fig S9). The plasmids may help the resistant bacteria share their ARGs with other strains, and the mobile and conjugative plasmids are particularly important in spreading ARGs in bacterial communities [11, 69]. Plasmids carrying shared ARGs were found in different genera in the same hospital [70]. Furthermore, the shared ARGs had a higher chance of being spread to the heat-tolerant hosts by HGTs than the chromosome-only ARGs; thus, they exhibited higher relative abundance in composts than the chromosome-only ARGs (Additional file 1: Fig. S9). We found that most (75.98%) of 204 identified ARG subtypes were related to shared contigs between plasmids and chromosome in our samples (Additional file 1: Fig. S8). This result differs from the results of the enrichment and metagenomic analyses of cow manure, which indicated that most ARGs were harbored on the chromosome [70], and also different from the results that plasmid-carried ARGs were dominant in metagenomic analysis of sewage waters [71] and wastewater treatment plants [72]. The multidrug resistance genes, particularly the major facilitator superfamily antibiotic efflux pump genes, were harbored on Enterobacteriaceae such as *Escherichia* and *Klebsiella*. These gene for cell wall/membrane/envelope biogenesis functions were often harbored by chromosomes than by plasmids, so less mobile ability maybe cause they could be remove efficiently after composting [73]. The chloramphenicol exporter is a multidrug resistance gene; however, all the chloramphenicol exporter carried ARC were linked with plasmids in our samples. Although the relative abundance of chloramphenicol exporter decreased significantly after composting, it still revealed a high average relative abundance in all ARG subtypes. Nevertheless, the MGEs are not the only reason for ARGs to escape composting; the relative abundance of a vancomycin resistance gene (*vanR*), which was not linked to any MGEs and only harbored on the chromosome, was significantly increased after composting owing to its 15 hosts, including thermophilic *Actinobacteria* (*Thermobispora*) and heat-tolerant *Firmicutes* (*Bacillus*) (Additional file 1: Table S5). However, *vanR* is an OmpR-family transcriptional activator in the *VanSR* regulatory system and could not represent vancomycin resistance independently [74], and in our samples *vanR* only carried by the chromosome, so the increase of *vanR* maybe not indicate the increase of vancomycin resistance in composts.

Based on the taxon classification of the MGE-related ARCs, we found that the dominant hosts of transposase-, integron-, and recombinase-related ARCs belonged to different phyla (Additional file 1: Fig. S11). Most integron-related ARCs belonged to *Proteobacteria*, which indicates that integrons mainly contributed to shared ARGs in *Proteobacteria* [33]. Transposase-related ARCs included all four phyla; Jiang et al. reported that Proteobacteria pathogens could acquire the ARGs from *Actinobacteria* with the transposon and conjugative plasmids [75]. Metagenomic analysis revealed that a majority (63.2%) of the recently transferred ARGs between plasmids and bacterial chromosomes can be attributed to the insertion sequences (ISs) [11]. Transposases are coded by ISs, and in our samples, 14.53% of the ARCs carried transposase (ISs) with 18 ARG types, whereas 7.52% carried integron elements with 13 ARG types. Thus, transposases (ISs) could play a pivotal role in mediating the transfer of ARGs between different phyla in animal manure and composting.

Compared to the read-based analysis, the assembly-based analysis may have lost some low-richness ARGs. In our samples, all the assembled 1200 ARCs (> 500 bp) covered 224 ARG subtypes, which only accounted for 35.8% of all 626 ARG subtypes based on the read analysis; however, the assembled ARCs accounted for 72.6% of the ARG subtypes of all the manure common resistome, which includes 201 ARG subtypes. Based on metagenomic analysis, we can directly obtain the mobility and host-related information of ARGs at the DNA level; however, further host tracking technologies (such as fluorescence-activated cell sorting or single cell fusion PCR) are required to verify the host changes of ARGs and HGTs during composting.

## Conclusion

The results obtained in the study showed commercial composting can significantly reduce most ARGs of common antibiotic resistome in different animal manure except for multidrug, sulfonamide, and trimethoprim resistance genes. Meanwhile, the host tracking and quantifying analysis suggested that the hosts of ARGs in manure have preference on animal species and the surviving ARGs in composts such as *sul1* and *aadA*, usually have a broad range of bacterial hosts and are also linked with most MGEs. Overall, our results indicated that composting can mitigate common resistome in animal manure by changing their bacterial hosts; however, some ARGs can escape composting with the survivor heat-tolerant hosts or transfer to these hosts. Thus, it is necessary to improve the commercial composting process to mitigate HGTs and curb the growth of heat-tolerant hosts in order to efficiently control the animal source ARGs.

## Supplementary Information

**Additional file 1. Supplementary Information. Supplementary methods. Fig. S1** The metagenomic analysis workflow in this study. **Fig. S2** Sampling map. **Fig. S3** Box-plot of ARG types relative abundance and numbers in manure and compost. **Fig. S4** Significantly higher relative abundance ARG subtypes (0.001 copies per 16S rRNA gene up) in compost samples. **Fig. S5** The top ten ARGs-hosts at genus level in manure and composts. **Fig. S6** The major hosts (top ten genera with average relative abundances in each sample) for M-L-S, tetracyclines, aminoglycosides, chloramphenicols and sulfonamides resistance genes. **Fig. S7** The alpha diversity analysis of ARG hosts in manure and composts. **Fig. S8** The bipartite network depicting the shared and unique ARGs between plasmid and chromosome. **Fig. S9** Variations of contigs carried common resistome in chromosomes and plasmids. **Fig. S10** The bipartite network showing the shared and unique ARGs with transponsase, integron and recombinase. **Fig. S11** Distribution of ARCs taxonomy in MGEs. **Fig. S12** Principal coordinate analysis based on relative abundance of bacteria at genus level. **Fig. S13** The heatmap on relative abundance of ARGs-contigs with integron. **Fig. S14** The heatmap on relative abundance of ARGs-contigs with transponsase. **Fig. S15** The heatmap on relative abundance of ARGs-contigs with recombinase. **Fig. S16** Co-occurring sul1 and MGEs and the relative abundance (cpm) of sul1-carrying contigs in manure and composts. **Fig. S17** The heatmap of relative abundance of PARB contigs. **Table S1** Sample information. **Table S2** The average relative abundance of ARG types in manure and compost. **Table S3** The taxonomy of ARCs at phylum level. **Table S4** Top 20 host bacteria of ARCs and carried ARG subtypes at genus level. **Table S5** The host bacteria of top 20 ARG subtypes. **Table S6** The number and percentage of MGEs-carried ARCs.

**Additional file 2. Dataset 1** Concentrations of antibiotics in manures and composts. Nineteen antibiotics including: sulfadiazine (SDZ), sulfadimethoxine (SMX), sulfamethoxazole (SMZ), sulfachlorpyridazine (SCP), tetracycline (TC), chlortetracycline (CTC), oxytetracycline (OTC), doxycycline (DC), erythromycin (ETM), tylosin (TYL), roxithromycin (RTM), norfloxacin (NFX), ciprofloxacin (CFX), enrofloxacin (EFX) and lomefloxacin (LFX), florfenicol (FF) and chloramphenicol (CAP), ceftiofur (CEFT).

**Additional file 3. Dataset 2** Bacterial community compositions in manure and compost samples at phylum level.

**Additional file 4.** Reads quality control results and taxonomy of antibiotic resistant contigs. **Dataset 3** Multiqc results of sequenced reads. **Dataset 4** The classification results of antibiotic resistant contigs by CAT and Kraken. **Dataset 5** LCA mapping each k-mer.

## Data Availability

All data generated during this study are available at the Genome Sequence Archive (GSA) repository under BioProject Number CRA005191.
