# Supplementary material for: Metagenomic assembly reveals hosts and mobility of common antibiotic resistome in animal manure and commercial compost
Source: Environ Microbiome. 2022 Aug 11;17:42. doi: 10.1186/s40793-022-00437-x (PMC9367140; doi:10.1186/s40793-022-00437-x)
Supplement: Supplementary file 1 — Additional file 1. Supplementary Information. Supplementary methods. Fig. S1 The metagenomic analysis workflow in this study. Fig. S2 Sampling map. Fig. S3 Box-plot of ARG types relative abundance and numbers in manure and compost. Fig. S4 Significantly higher relative abundance ARG subtypes (0.001 copies per 16S rRNA gene up) in compost samples. Fig. S5 The top ten ARGs-hosts at genus level in manure and composts. Fig. S6 The major hosts (top ten genera with average relative abundances in each sample) for M-L-S, tetracyclines, aminoglycosides, chloramphenicols and sulfonamides resistance genes. Fig. S7 The alpha diversity analysis of ARG hosts in manure and composts. Fig. S8 The bipartite network depicting the shared and unique ARGs between plasmid and chromosome. Fig. S9 Variations of contigs carried common resistome in chromosomes and plasmids. Fig. S10 The bipartite network showing the shared and unique ARGs with transponsase, integron and recombinase. Fig. S11 Distribution of ARCs taxonomy in MGEs. Fig. S12 Principal coordinate analysis based on relative abundance of bacteria at genus level. Fig. S13 The heatmap on relative abundance of ARGs-contigs with integron. Fig. S14 The heatmap on relative abundance of ARGs-contigs with transponsase. Fig. S15 The heatmap on relative abundance of ARGs-contigs with recombinase. Fig. S16 Co-occurring sul1 and MGEs and the relative abundance (cpm) of sul1-carrying contigs in manure and composts. Fig. S17 The heatmap of relative abundance of PARB contigs. Table S1 Sample information. Table S2 The average relative abundance of ARG types in manure and compost. Table S3 The taxonomy of ARCs at phylum level. Table S4 Top 20 host bacteria of ARCs and carried ARG subtypes at genus level. Table S5 The host bacteria of top 20 ARG subtypes. Table S6 The number and percentage of MGEs-carried ARCs. [file 40793_2022_437_MOESM1_ESM.docx]

# Supplementary Information for

# Metagenomic Assembly Reveals Hosts and Mobility of Common Antibiotic Resistome in Animal Manure and Commercial Compost

Tianlei Qiu^a^, Linhe Huo^a^, Yajie Guo^a^, Min Gao^a^, Guoliang Wang^a^, Dong Hu^b^, Cheng Li^c^, Zhanwu Wang^b^, Guiming Liu^a*^, Xuming Wang^a^*

^a^Beijing Key Laboratory of Agricultural Genetic Resources and Biotechnology, Institute of Biotechnology, Beijing Academy of Agriculture and Forestry Sciences, Beijing 100097, China

^b^Institute of Agro-resources and Environment (Hebei Fertilizer Technology Innovation Center), Hebei Academy of Agriculture and Forestry Sciences, Shijiazhuang, Hebei, P. R. China

^c^Institute of Quality Standardand Testing Technology, Beijing Academy of Agriculture and Forestry Sciences, Beijing 100097, China

*To whom correspondence should be addressed. E-mail: wangxuming@baafs.net.cn

## Figure and table legends

**Supplementary methods**

## Figure S1 The metagenomic analysis workflow in this study.

**Figure S2** Sampling map

**Figure S3** Box-plot of ARG types relative abundance and numbers in manure and compost.

**Figure S4** Significantly higher relative abundance ARG subtypes (0.001 copies per 16S rRNA gene up) in compost samples.

**Figure S5** The top ten ARGs-hosts at genus level in manure and composts.

**Figure S6** The major hosts (top ten genera with average relative abundances in each sample) for M-L-S, tetracyclines, aminoglycosides, chloramphenicols and sulfonamides resistance genes.

**Figure S7** The alpha diversity analysis of ARG hosts in manure and composts.

**Figure S8** The bipartite network depicting the shared and unique ARGs between plasmid and chromosome.

**Figure S9** Variations of contigs carried common resistome in chromosomes and plasmids.

**Figure S10** The bipartite network showing the shared and unique ARGs with transponsase, integron and recombinase.

**Figure S11** Distribution of ARCs taxonomy in MGEs.

**Figure S12** Principal coordinate analysis based on relative abundance of bacteria at genus level.

**Figure S13** The heatmap on relative abundance of ARGs-contigs with integron.

**Figure S14** The heatmap on relative abundance of ARGs-contigs with transponsase.

**Figure S15** The heatmap on relative abundance of ARGs-contigs with recombinase.

**Figure S16** Co-occurring sul1 and MGEs and the relative abundance (cpm) of sul1-carrying contigs in manure and composts.

**Figure S17** The heatmap of relative abundance of PARB contigs.

**Table S1** Sample information

**Table S2** The average relative abundance of ARG types in manure and compost.

**Table S3** The taxonomy of ARCs at phylum level.

**Table S4** Top 20 host bacteria of ARCs and carried ARG subtypes at genus level.

**Table S5** The host bacteria of top 20 ARG subtypes.

**Table S6** The number and percentage of MGEs-carried ARCs.

## Supplementary methods

## Sample collection

Manure and compost samples were collected from 29 concentrated animal feeding operations farms and 13 commercial manure composting plants from Beijing and Hebei Province, Northern China (representative sites; including eight broiler farms, eight layer farms, five swine farms, four beef cow farms, and four dairy cow farms in northern China) (Fig. S1 and Table S1). Sampling campaigns were conducted between October 2017 and October 2018. Fresh manure samples (500 g) were collected by mixing subsamples directly from five different points of each farm to ensure representativeness. Using the same sampling methods, 500 g of compost samples were collected from the finished compost warehouses. All samples were incubated on ice in coolers, transported immediately to the laboratory for pretreatment, and then stored at –80 °C until further analysis.

## Determination of antibiotic concentration

The 19 antibiotics analyzed in this work belong to five categories and were mainly selected from those used for livestock and poultry animals. They included four tetracyclines (tetracycline, oxytetracycline, chlortetracycline, and doxycycline), four sulfonamides (sulfadiazine, sulfamethoxazole, sulfamethoxazole, and sulfachlorpyridazine), four quinolones (norfloxacin, ciprofloxacin, enrofloxacin, and lomefloxacin), three macrolides (erythromycin, tylosin, and roxithromycin), and two amphenicols (florfenicol and chloramphenicol). Two other antibiotics included lincomycin and ceftiofur. Antibiotics were pretreated and analyzed according to Li et al.’s study([Li, Li et al. 2020](#_ENREF_7)).

## DNA extraction, metagenomic sequencing, and quality analysis

DNA was extracted from three replicates of each manure or compost sample using the MoBio PowerSoil DNA isolation kit (Mobio Laboratories, Carlsbad, CA, USA) according to the manufacturer’s protocol. The concentration and quality of the extracted DNA were determined using Nanodrop 2000c and Qubit 3.0 Fluorometer (Thermo Fisher Scientific, DE, USA). The DNA from the manure and compost samples was used for shotgun sequencing using the Illumina Novaseq 6000 and paired-end 150 bp reads were generated. The raw data obtained from the Illumina HiSeq sequencing platform using Readfq V8 (https://github.com/cjfields/readfq) was preprocessed to acquire clean data for subsequent analyses. The specific processing steps were as follows: a) removal of reads comprising low quality bases (quality threshold value ≤38) >40 bp; b) removal of reads wherein the N base reached 10 bp; c) removal of reads presenting an overlap >15 bp with Adapter. On average, 23.36 M (SD ± 6.36 M) reads passed and were used in all further analyses. The corresponding quality control of all reads were checked by fastqc and multiqc. The quality report and depth of each sample were added in the Additional file 4 Dataset 3. Sequencing data were deposited in the Genome Sequence Archive repository under BioProject number CRA005191.

## Calculating ARG abundance

ARG abundance was determined using ARGs-OAP v2.0, which integrates the detection of ARGs using the reference database SARG.2.2([Yin, Jiang et al. 2018](#_ENREF_16)). Briefly, reads were annotated as ARG-like reads at the E-value cutoff of 10^-7^, sequence identity of 90%, and alignment length of more than 25 amino acids. ARGs were quantified by normalizing ARG abundance to the copy number of the 16S rRNA gene using the following equation([Yang, Jiang et al. 2016](#_ENREF_15)):

$$Abundance=\sum_{1}^{n} \frac{N_{ARG-like sequence}\times L_{reads}/L_{ARG reference seqeuence}}{N_{16S sequence}\times L_{reads}/L_{16S sequence}}$$

where $N_{ARG-like sequence}$ is the number of ARG-like sequences annotated to one specific ARG reference sequence, $L_{reads}$ represents the length of the reads, $L_{ARG reference seqeuence}$ is the nucleotide sequence length of the corresponding ARG reference sequence, $N_{16S sequence}$ is the number of the 16S rRNA gene sequences, $L_{16S sequence}$ is the full length of the 16S rRNA gene, and $n$ is the number of mapped ARG reference sequences belonging to the ARG type or subtype.

The Tukey HSD test was conducted in R to analyze the statistical differences between different animal manures and composts. The t-test or wilcox.test in R were used to analyze the statistical differences between manure and compost. The correlation matrix was constructed with ARG and antibiotics by calculating all pairwise Spearman’s rank correlations. A correlation between any two items was considered statistically significant if the Spearman’s correlation coefficient (ρ) > 0.5 and *p* < 0.05. The resulting correlation matrixes were translated into an association network using Gephi 0.9.2([Bastian, Heymann et al. 2009](#_ENREF_1)). The protest in the R vegan package was conducted to Procrustes between antibiotic residues and ARGs in all samples.

## Metagenomic data analysis

First, Kraken2 was used to obtain the taxonomic profile of each sample, and its relative abundance was estimated using Bracken based on filtered metagenomic data([Wood and Salzberg 2014](#_ENREF_14), [Lu, Breitwieser et al. 2016](#_ENREF_9)). Principal coordinate analysis (PCoA) was used to estimate the community dissimilarities based on the bacterial community structure at the genus level using the vegan package in R.

Metagenomes were assembled de novo for each sample using MEGAHIT([Li, Liu et al. 2015](#_ENREF_8)); thereafter, all contigs were combined into a single file. All contigs over 500 bp in the combined file were retained and merged at 95% identity using CD-HIT-EST([Fu, Niu et al. 2012](#_ENREF_6)). An 8.11 M non-redundant contigs longer than 500 bp were obtained including 2.60 M contigs longer than 1000 bp. The N50 of the non-redundant contigs was 1723 bp and the L50 was 1 150 602 bp. For identifying antibiotic resistance contigs (ARCs), the DIAMOND (v0.9.22.123) was used to search the protein sequences of the SARG.2.2 database using BLASTx algorithm with an E-value cutoff of 1e^-5^. The best hit results were filtered with an identity cutoff of 80% and a subject (ARGs) coverage cutoff of 70%. Based on the results of BLASTx, there were 1356 ARCs were matched to the SARG 2.2 database. For the multiple ARGs annotated, genes were predicted on the ARGs using Prokka([Seemann 2014](#_ENREF_12)). ARG-like open reading frames (ORFs) on ARCs were determined using BLASTP against the SARG.2.2 database with a minimum similarity of 80% over 70% of the query coverage([Ma, Xia et al. 2016](#_ENREF_10)). Based the reannotated results of BLASTP, the number of non-redundant ARCs were revised to 1200.

The occurrence of ARCs in chromosomes or plasmids was determined using BLASTn against NCBI Ref of 14827 complete bacterial genome and 33580 complete plasmid (until July 2021). Hit ARCs with >80% identity and >70% query coverage were retained.

For MGEs, one ORF was considered as a transposase or recombinase gene if one of the following keywords was in its best BLAST hit description: transposase, transposon, or recombinase ([Forsberg, Patel et al. 2014](#_ENREF_4)). Integrons in ARCs were identified using the IntegronFinder ([Cury, Jové et al. 2016](#_ENREF_3)).

The virulence genes were identified using Abricate (https://github.com/tseemann/abricate) by comparing ARCs against VFdb ([Chen, Xiong et al. 2011](#_ENREF_2)). Only hits with sequence identity >70% and query coverage >90% were retained ([Fresia, Antelo et al. 2019](#_ENREF_5)).

The abundance of non-redundant contigs in each sample was determined using Salmon ([Patro, Duggal et al. 2017](#_ENREF_11)). The relative abundance of contigs was presented with contigs per kilobase per million mapped reads (cpm) according to the following equation:

$$cpm=1,000,000*\frac{{reads mapped to contig}/{contig length}}{sum({reads mapped to contig}/{contig length})}$$

The relative abundance of ARCs was extracted from the whole quantification table of all non-redundant contigs. The ARCs were first taxonomically classified using Kraken2 in metaWARP ([Uritskiy, DiRuggiero et al. 2018](#_ENREF_13)) (Additional files 4. Dataset 4 and 5).

The relative abundance of ARG hosts at the phylum or genus level were calculated by summing the cpm of ARCs under the specific taxonomic group. PCoA based on the ARG hosts at the genus level was used to estimate the community dissimilarities using the vegan package in R. Bipartite network analysis was used to uncover the unique and shared ARG subtypes in plasmids and chromosomes and analyze ARG subtypes with integron, transposase, and recombinase. Gephi software was used to visualize the bipartite and ARG host networks.

## Figure S1 The metagenomic analysis workflow in this study.


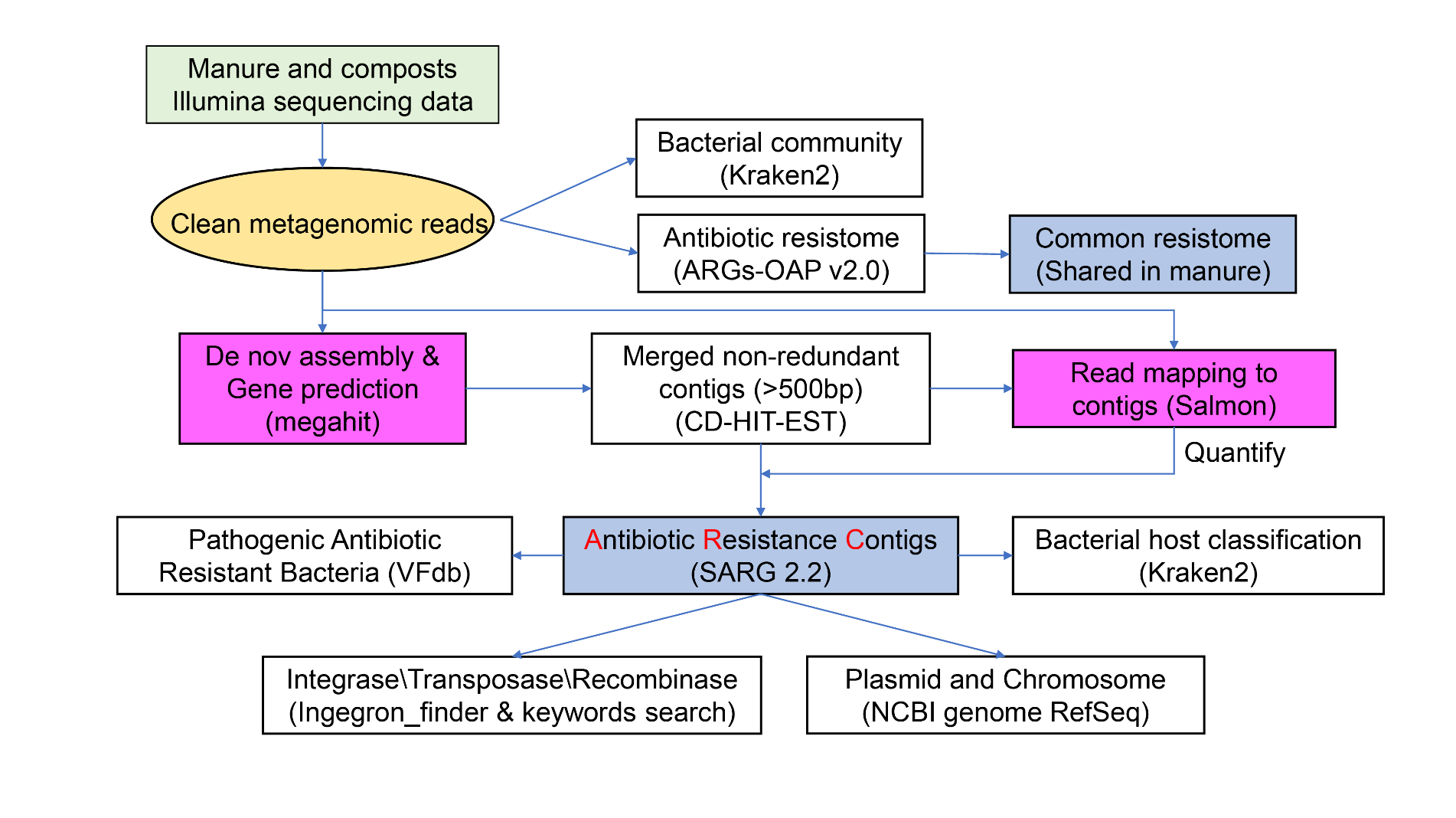


## Figure S2 Sampling map


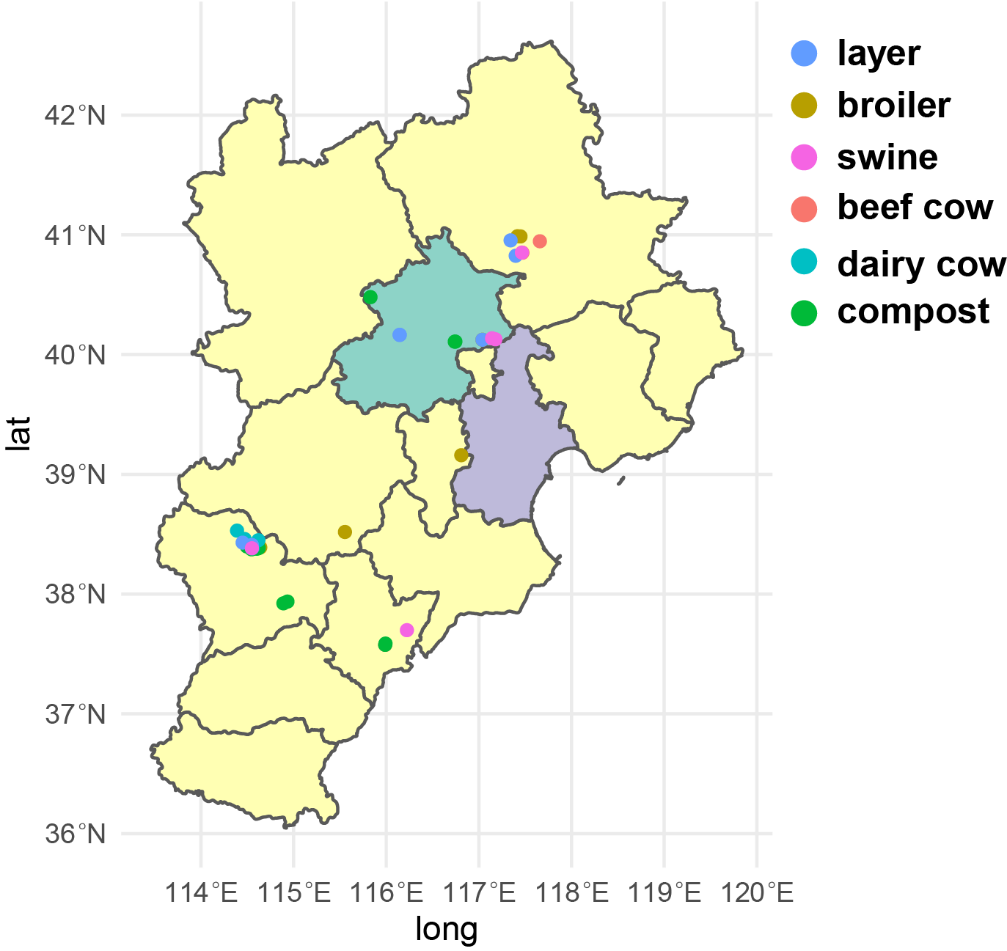


## Figure S3 Box-plot of ARG types relative abundance and numbers in manure and compost


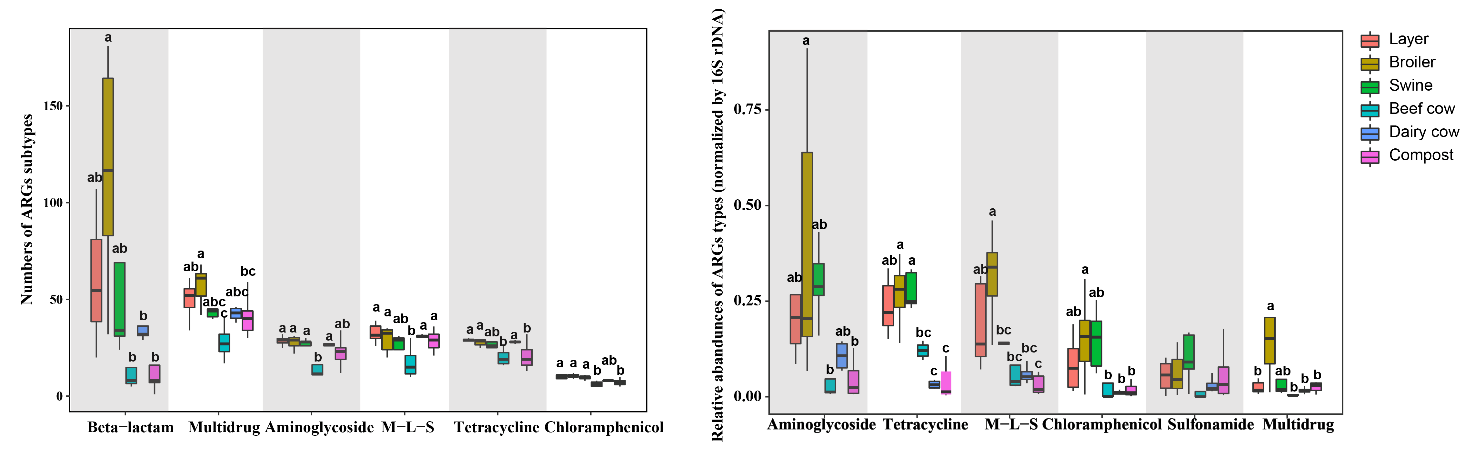


## Figure S4 Significantly higher relative abundance ARG subtype (0.001 copies per 16S rRNA gene up) in compost samples.


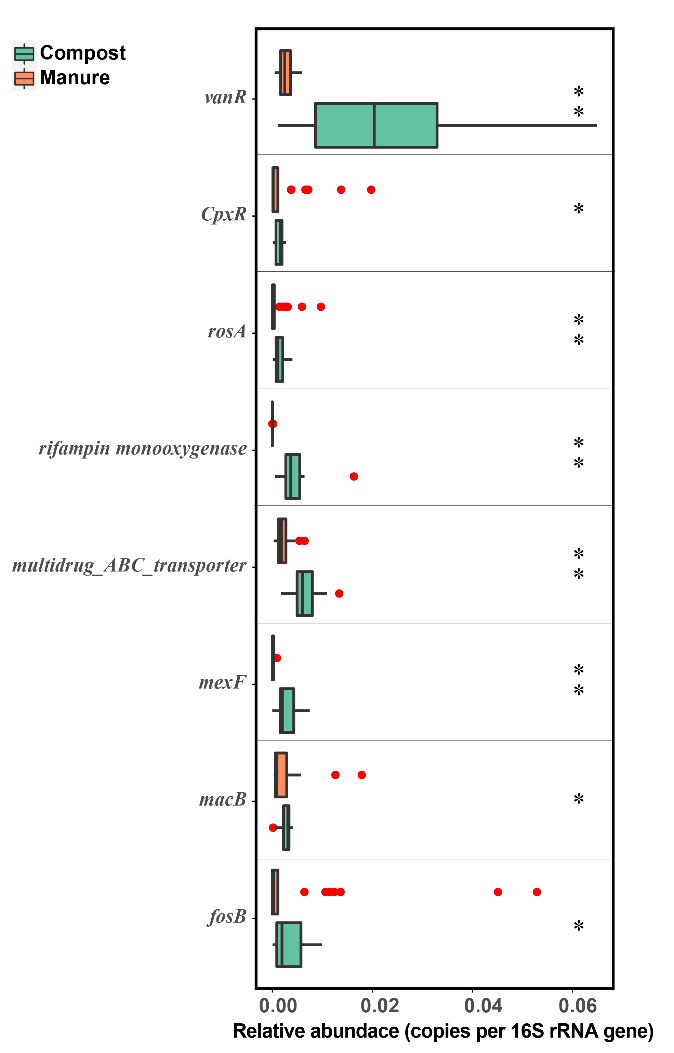


## Figure S5 The top ten ARGs-hosts at genus level based on the relative abundance of ARCs in manure and composts


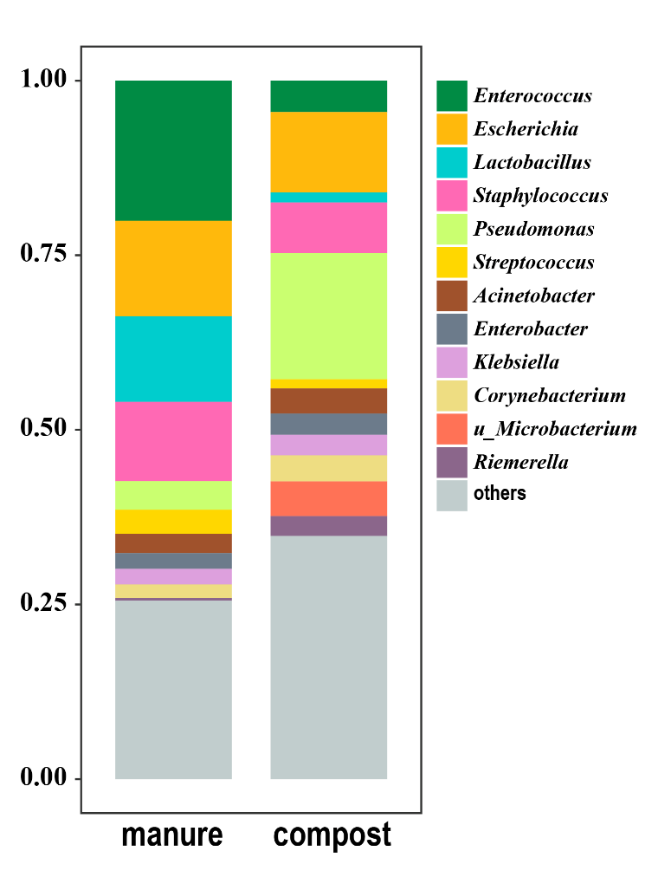


## Figure S6 The major hosts (top ten genera with average relative abundances in each sample) for M-L-S, tetracyclines, aminoglycosides, chloramphenicols and sulfonamides resistance genes.





## Figure S7 The alpha diversity analysis of ARG hosts in manure and composts.

(A) Shannon index; (B) Simpson index; (C) Richness index; (D) Ace index; (E) Chao1 index; (F) Pielou index. The letters a and b identify groups that differ significantly (p < 0.05).


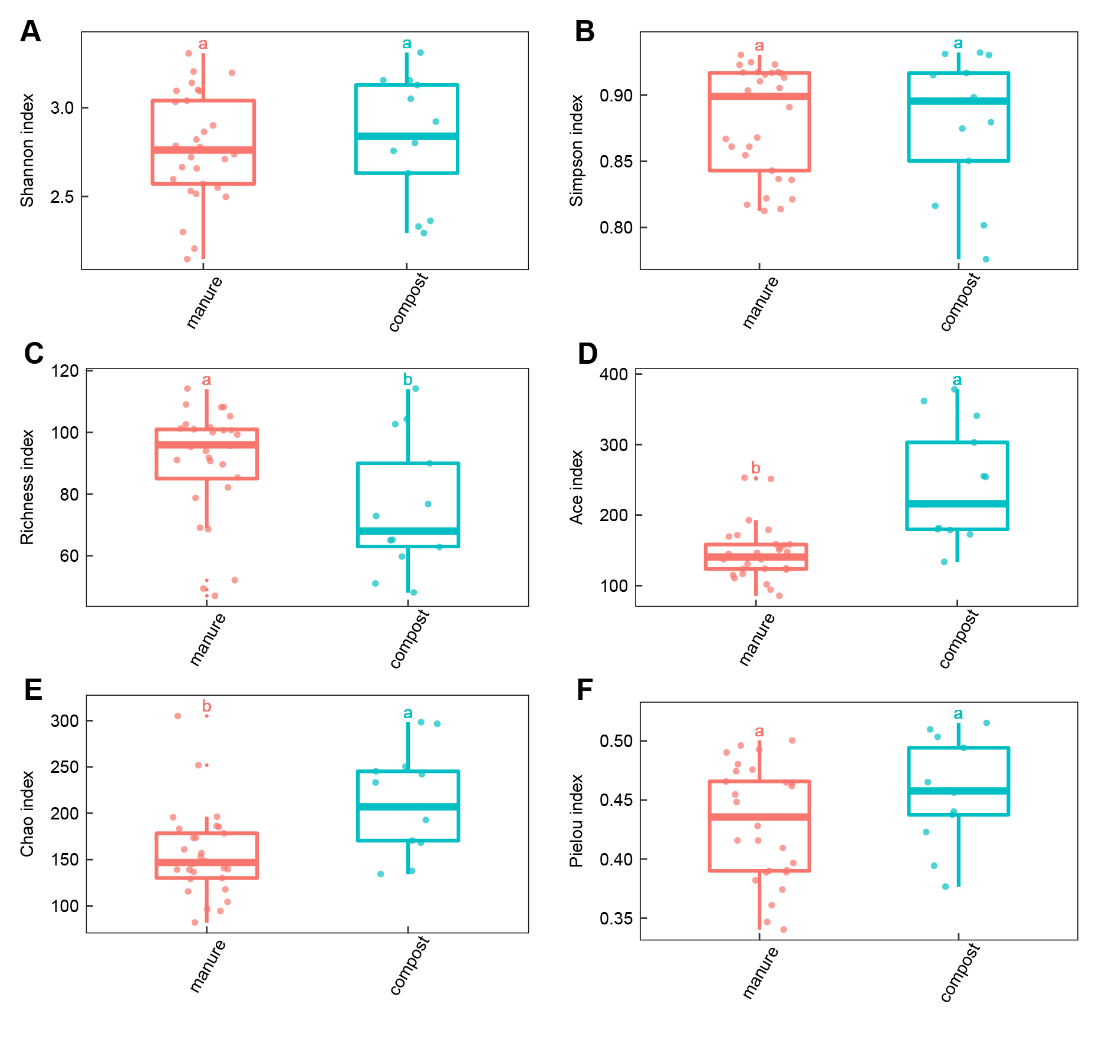


## Figure S8 The bipartite network depicting the shared and unique ARGs between plasmid and chromosome. The nodes were colored according to ARG types. M-L-S: macrolidelincosamide–streptogramin. The most abundant ARG subtypes (top20) in manure common resistome are shown in red letters.


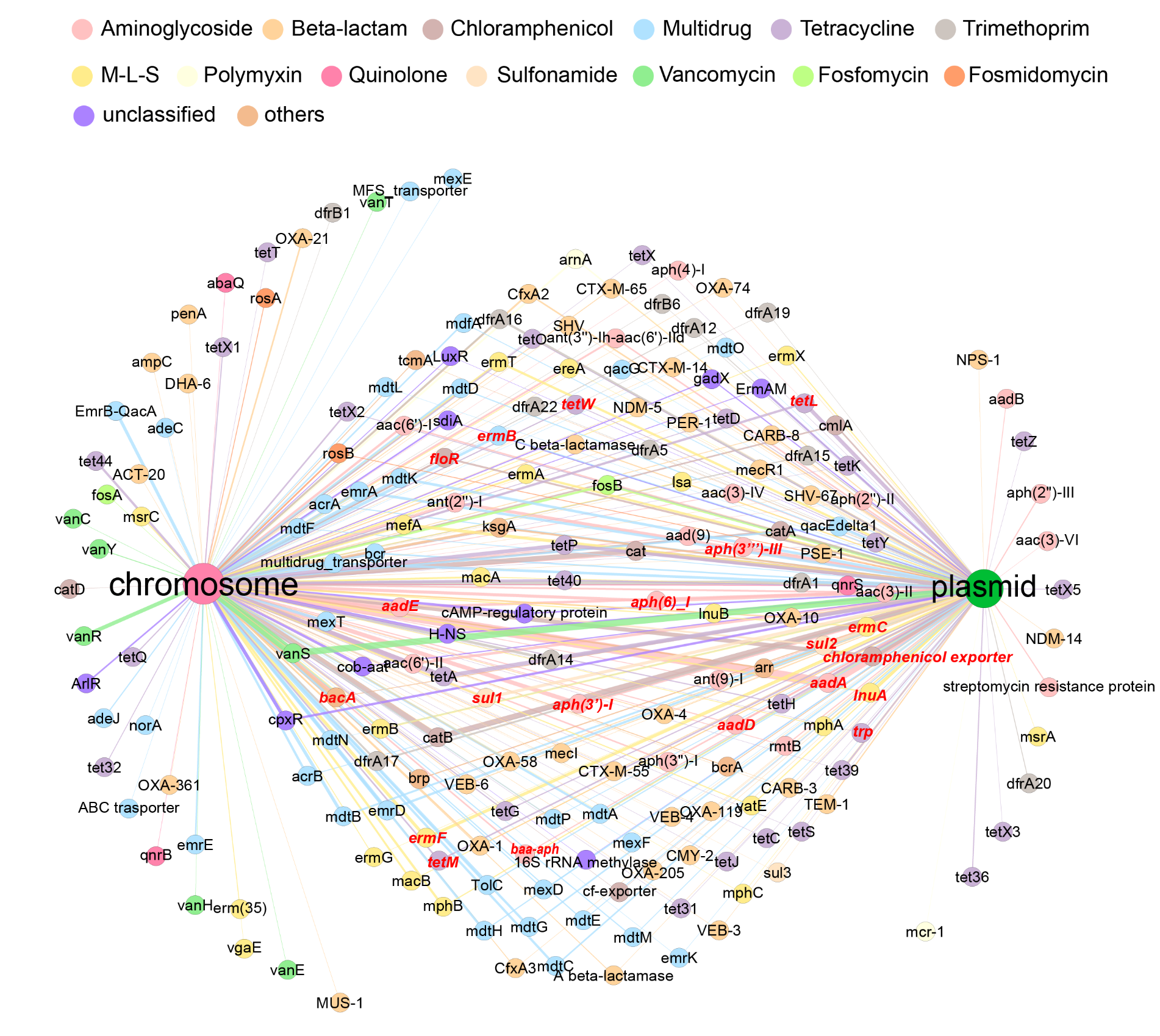


## Figure S9 Variations of contigs carried common resistome in chromosomes and plasmids. The ARCs, wherein relative abundance was greater than 5 cpm, were used. The color of the square on the left shows the type of ARGs carried on each ARC. If it carries more than 1 ARGs, it will be displayed mixed, and the row name on the right will be marked in red. The row names are in order (ARG subtypes: Taxon: Seq.ID).


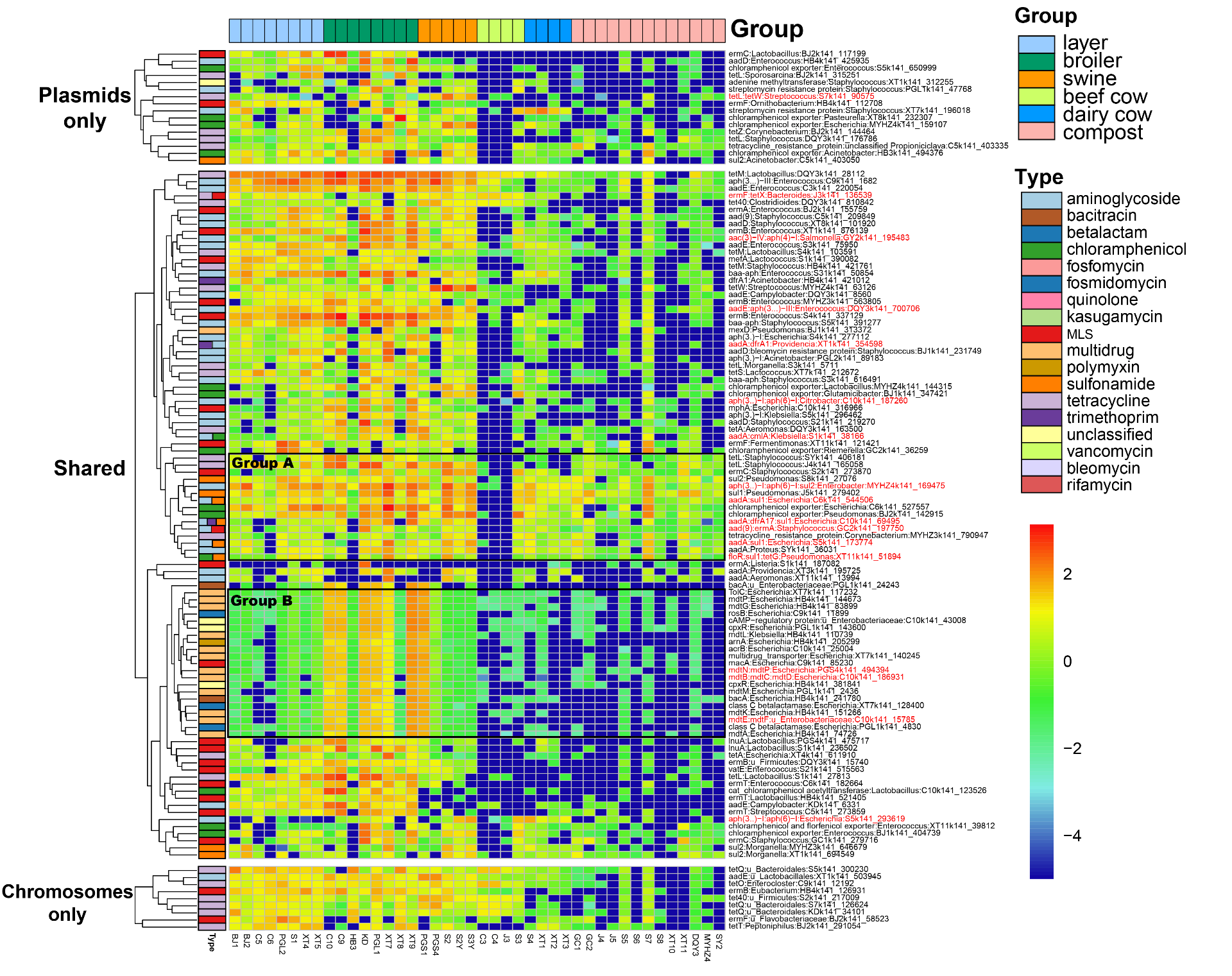


## Figure S10 The bipartite network showing the shared and unique ARGs with transponsase, integron and recombinase. The nodes were colored according to ARG types. M-L-S: macrolidelincosamide-streptogramin.


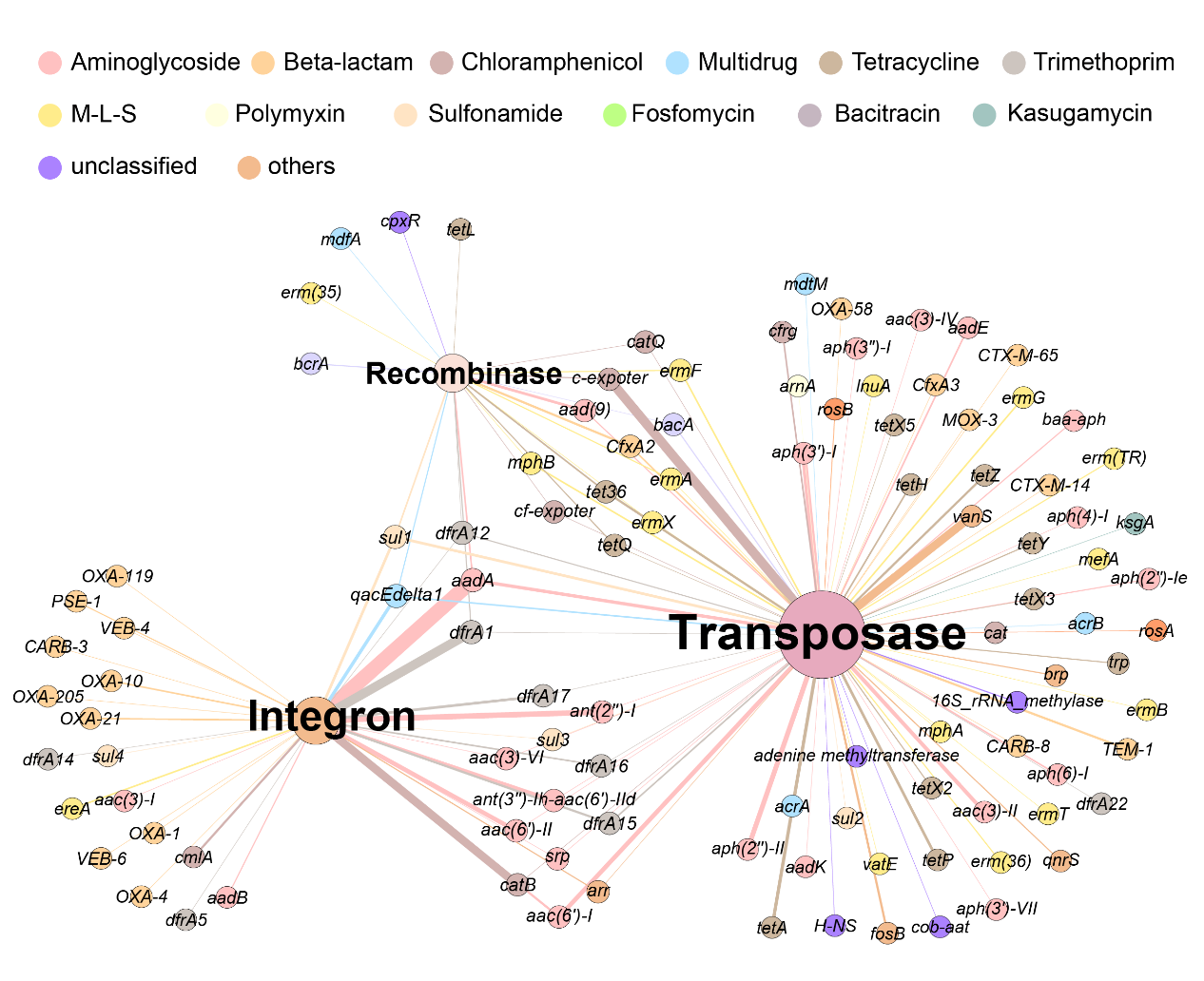


## Figure S11 Distribution of ARCs taxonomy in MGEs.

Distribution of MGEs（transponsase, integron, recombinase）carried ARCs taxon

Figure produced using the circlize package in R(4.0.2).


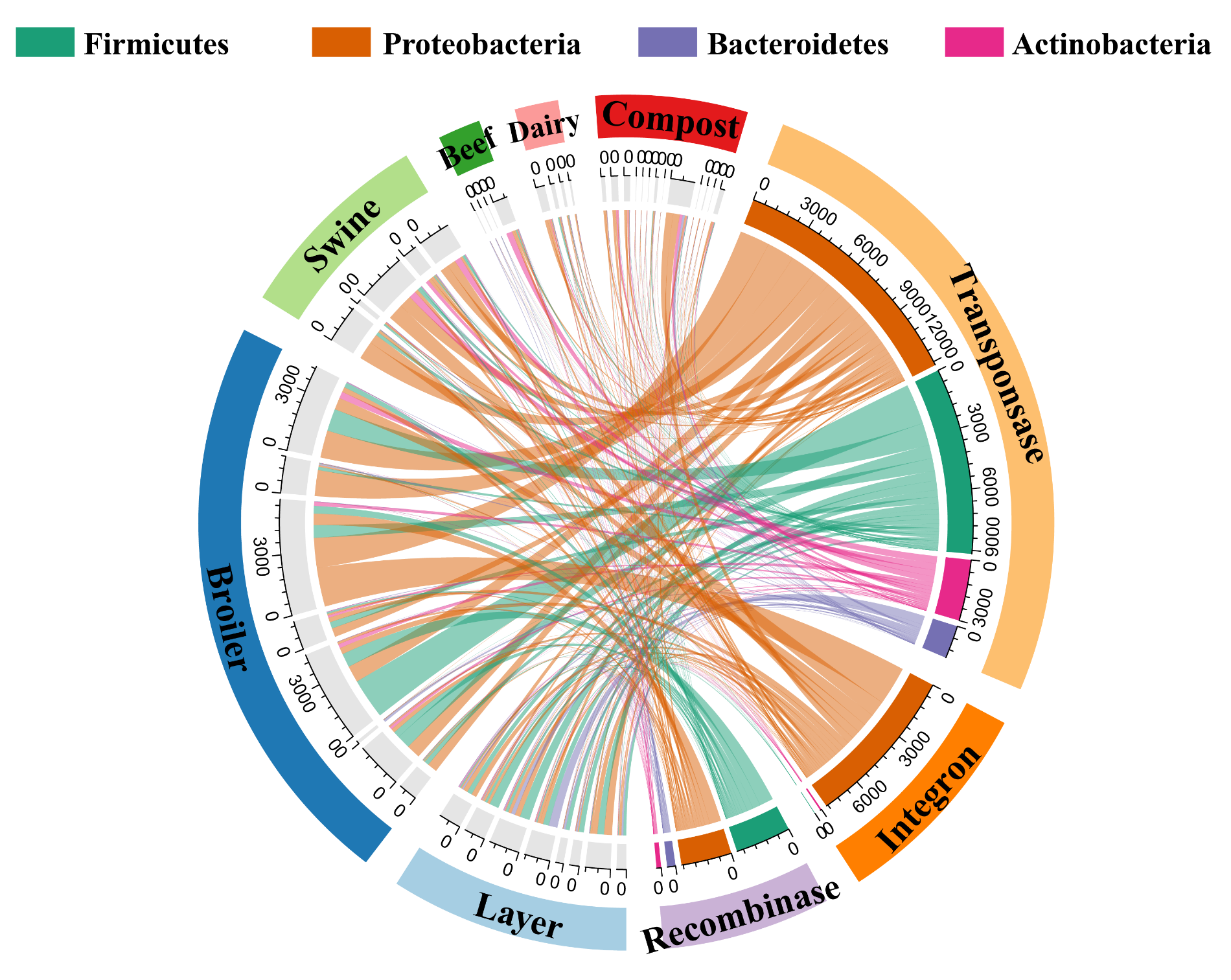


## Figure S12 Principal coordinate analysis based on relative abundance of bacteria at genus level. a. the total bacteria; b. the ARG hosts


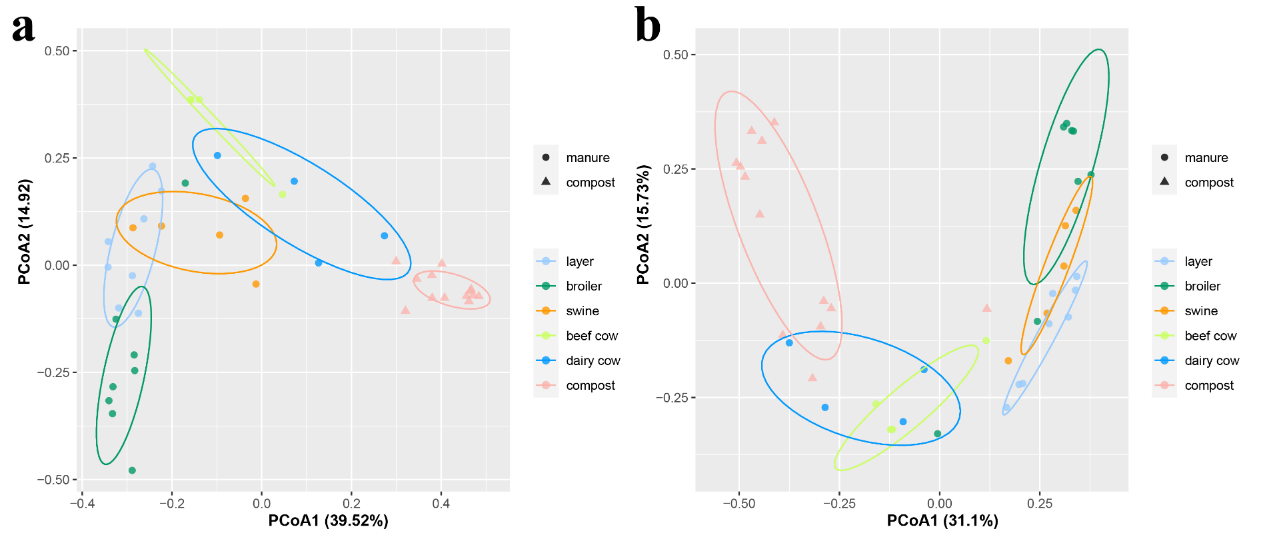


## Figure S13 The heatmap on relative abundance of ARGs-contigs with integron

The color of the square on the left shows the type of ARGs carried on each ARC. If it carries more than 1 ARGs, it will be displayed mixed, and the row name on the right will be marked in red; The row names are in order (ARG subtypes: Taxon: Seq.ID).


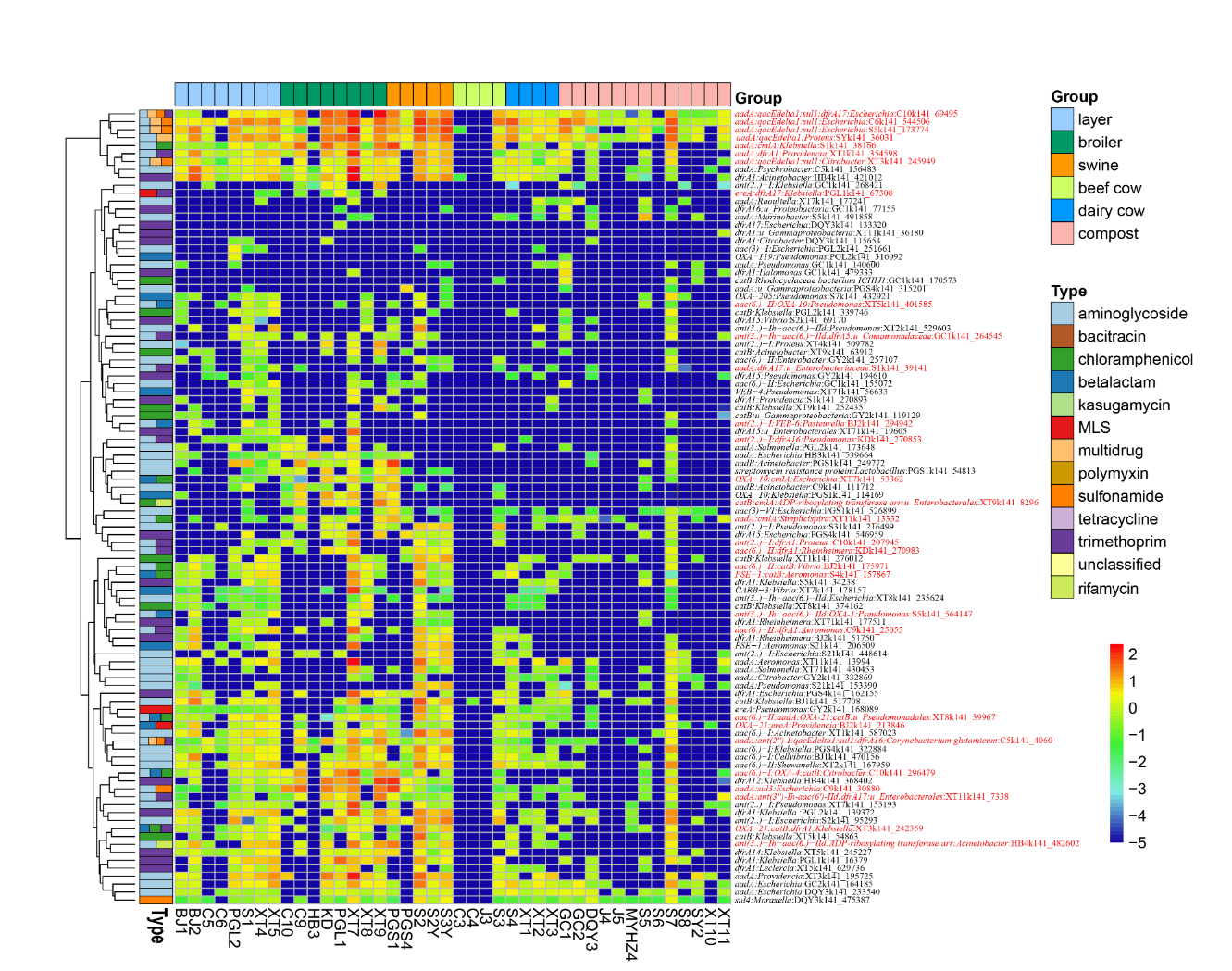


## Figure S14 The heatmap on relative abundance of ARGs-contigs with transponsase

The color of the square on the left shows the type of ARGs carried on each ARC. If it carries more than 1 ARGs, it will be displayed mixed, and the row name on the right will be marked in red; The row names are in order (ARG subtypes: Taxon: Seq.ID).


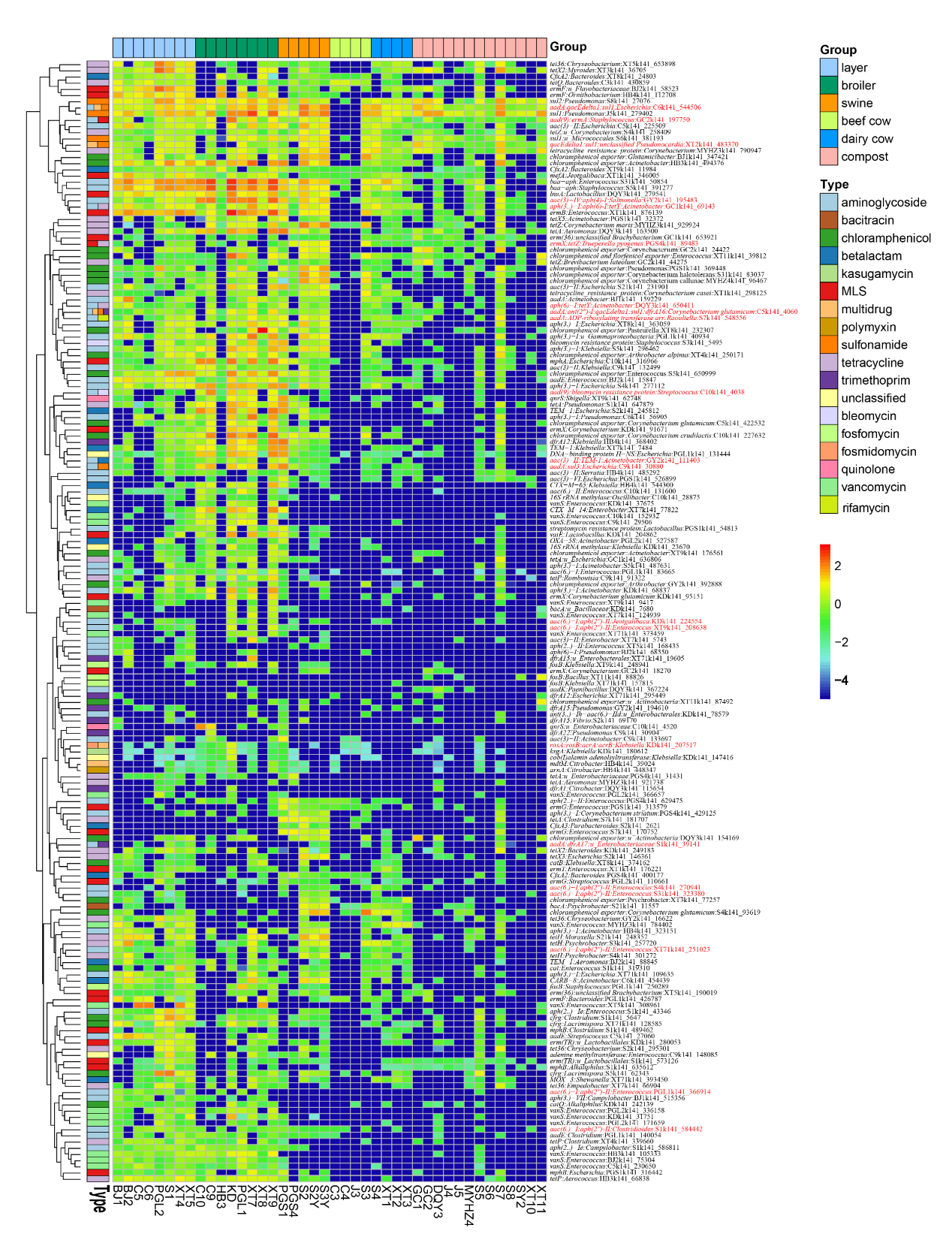


## Figure S15 The heatmap on relative abundance of ARGs-contigs with recombinase


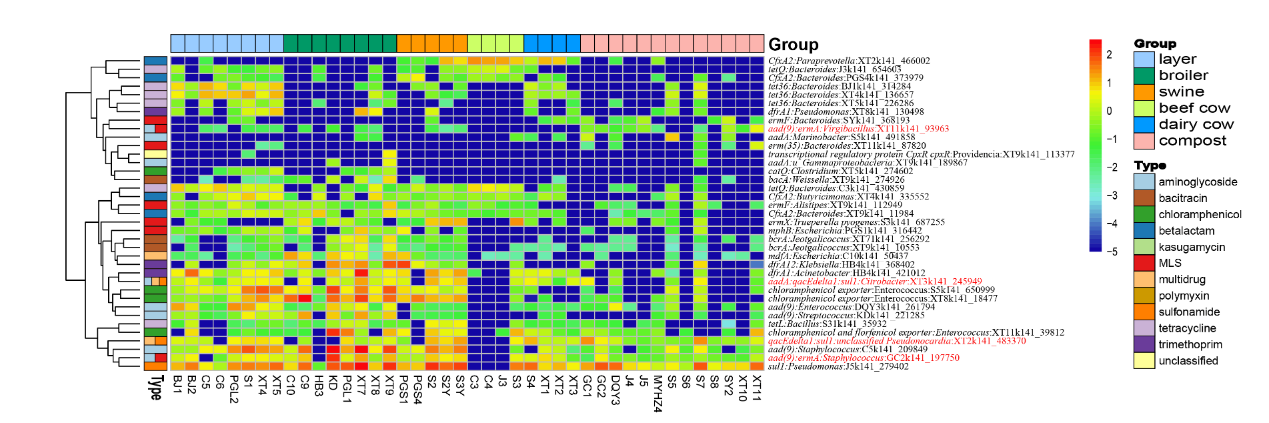


## Figure S16 Co-occurring sul1 and MGEs and the relative abundance (cpm) of sul1-carrying contigs in manure and composts.

a. the relative abundance (cpm) of *sul1*-carrying ARCs in manure and composts. b. the genetic environment of sul1 in manure and composts. Arrows indicate the directions of the genes, and different ARG types and MGEs are shown in different colors.


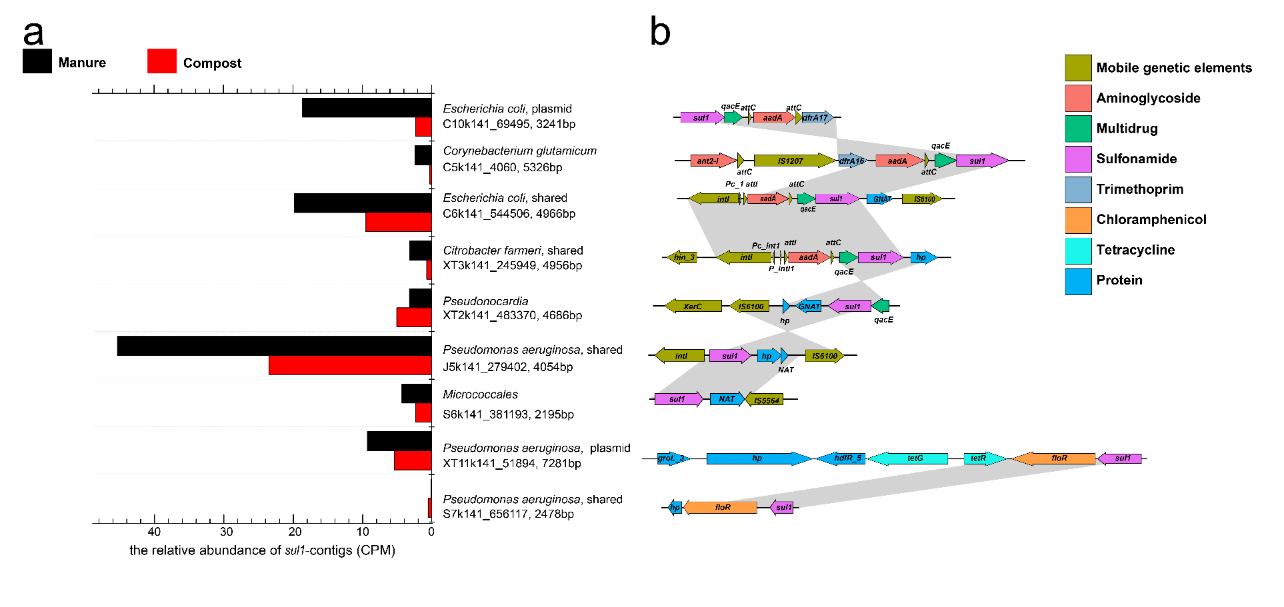


## Figure S17 The heatmap of relative abundance of potential pathogenic antibiotic-resistant bacteria

#Contigs located on the plasmids.

*Contigs carried transposase gene.


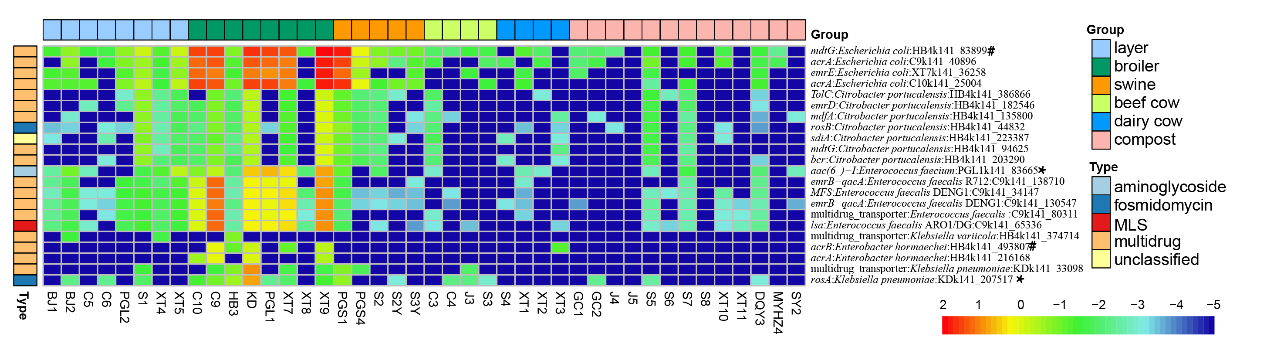


## Table S1 Sample information

|  | Sampling site | Sample type | Breeding scale | Days of age | Composting material |
| --- | --- | --- | --- | --- | --- |
| 1 | BJ1 | layer | 40000 | 250 |  |
| 2 | BJ2 | layer | 40000 | 400 |  |
| 3 | C5 | layer | 150000 | 220 |  |
| 4 | C6 | layer | 115000 | 250 |  |
| 5 | PGL2 | layer | 30000 | 160 |  |
| 6 | S1 | layer | 3000 | 300 |  |
| 7 | XT4 | layer | 200000 | 500 |  |
| 8 | XT5 | layer | 150000 | 600 |  |
| 9 | C10 | broiler | 28000 | 25 |  |
| 10 | C9 | broiler | 60000 | 25 |  |
| 11 | HB3 | broiler | 20000 | 18 |  |
| 12 | KD | broiler | 100000 | 29 |  |
| 13 | PGL1 | broiler | 30000 | 22 |  |
| 14 | XT7 | broiler | 60000 | 20 |  |
| 15 | XT8 | broiler | 80000 | 10 |  |
| 16 | XT9 | broiler | 50000 | 50 |  |
| 17 | C3 | beef cow | 510 | 300 |  |
| 18 | C4 | beef cow | 106 | 300 |  |
| 19 | J3 | beef cow | 1000 | -- |  |
| 20 | S3 | beef cow | 500 | 180 |  |
| 21 | PGS1 | swine | 1500 | -- |  |
| 22 | PGS4 | swine | 2000 | -- |  |
| 23 | S2 | swine | 1000 | 180 |  |
| 24 | S2Y | swine | 30000 | -- |  |
| 25 | S3Y | swine | 500 | 150 |  |
| 26 | S4 | dairy cow | 500 | 700 |  |
| 27 | XT1 | dairy cow | 800 | 900 |  |
| 28 | XT2 | dairy cow | 1200 | 1200 |  |
| 29 | XT3 | dairy cow | 1000 | 1350 |  |
| 30 | DQY3 | compost |  |  | layer manure, biogas dregs, corn stalks |
| 31 | GC1 | compost |  |  | layer manure, swine manure, mushroom residue |
| 32 | GC2 | compost |  |  | layer manure, swine manure, mushroom residue |
| 33 | J4 | compost |  |  | Beef cow manure, swine manure |
| 34 | J5 | compost |  |  | Dairy cow manure, swine manure |
| 35 | MYHZ4 | compost |  |  | Dairy manure, biogas dregs |
| 36 | S5 | compost |  |  | Layer manure |
| 37 | S6 | compost |  |  | Swine manure |
| 38 | S7 | compost |  |  | Beef cow manure, broiler manure |
| 39 | S8 | compost |  |  | Dairy cow manure |
| 40 | SY2 | compost |  |  | Beef cow manure, garden waste |
| 41 | XT10 | compost |  |  | Dairy cow manure, swine manure |
| 42 | XT11 | compost |  |  | Dairy cow manure, broiler manure |

## Table S2 The average relative abundance of ARG types in manure and compost.

| ARG types | Manure | | Compost | | *P*-value^#^ |
| --- | --- | --- | --- | --- | --- |
|  | Average RAs | Percent (%) | Average RAs | Percent (%) |  |
| Aminoglycoside | 0.237±0.215 | 25.2 | 0.0615±0.0985 | 17.7 | 0.00147** |
| Tetracycline | 0.206±0.102 | 21.9 | 0.0530±0.0803 | 15.2 | 0.000253** |
| M-L-S | 0.174±0.127 | 18.6 | 0.0336±0.0324 | 9.68 | 0.000157** |
| Chloramphenicol | 0.0947±0.0868 | 10.1 | 0.0389±0.0830 | 11.2 | 0.0329* |
| Sulfonamide | 0.0632±0.0764 | 6.74 | 0.0649±0.0874 | 18.7 | 0.5 |
| Multidrug | 0.0566±0.0881 | 6.03 | 0.0314±0.0242 | 9.04 | 0.666 |
| Beta-lactam | 0.0253±0.0186 | 2.69 | 0.00431±0.00866 | 1.24 | 0.0000521** |
| Unclassified | 0.0192±0.0169 | 2.04 | 0.0042±0.00380 | 1.21 | 0.000576** |
| Bacitracin | 0.0144±0.00886 | 1.54 | 0.00689±0.00273 | 1.98 | 0.00238** |
| Trimethoprim | 0.0123±0.0216 | 1.31 | 0.00494±0.0102 | 1.42 | 0.0668 |
| Vancomycin | 0.0119±0.00930 | 1.27 | 0.0268±0.0194 | 7.73 | 0.00587** |
| Bleomycin | 0.0079±0.0138 | 0.841 | 0.00129±0.00278 | 0.372 | 0.00818** |
| Fosfomycin | 0.00572±0.0131 | 0.610 | 0.00382±0.00333 | 1.10 | 0.017* |
| Rifamycin | 0.00252±0.00374 | 0.268 | 0.00677±0.00374 | 1.95 | 0.000096** |
| Fosmidomycin | 0.00225±0.00480 | 0.24 | 0.00275±0.00195 | 0.79 | 0.0151* |
| Kasugamycin | 0.00218±0.00431 | 0.232 | 0.00004±0.00005 | 0.0105 | 0.00355** |
| Polymyxin | 0.00182±0.00415 | 0.194 | 0.00002±0.00005 | 0.00682 | 0.00223** |
| Quinolone | 0.00137±0.00288 | 0.146 | 0.00095±0.00067 | 0.274 | 0.0182* |
| Total | 0.938±0.668 | 1.00 | 0.347±0.405 | 1.00 | 0.0016** |

#The asterisk “*”and “**” denote the significance level of 0.05 and 0.01, respectively.

## Table S3 The taxon of ARCs at phylum level

| Phylum | ARCs Number | Percentage (%) |
| --- | --- | --- |
| Proteobacteria | 602 | 50.08 |
| Firmicutes | 454 | 37.77 |
| Bacteroidetes | 78 | 6.49 |
| Actinobacteria | 63 | 5.24 |
| Fusobacteria | 4 | 0.33 |
| Synergistetes | 1 | 0.08 |

## Table S4 Top 20 host bacteria of ARCs and carried ARG subtypes at genus level.

| Taxon | ARCs | | Carried ARG subtype | |
| --- | --- | --- | --- | --- |
|  | number | Percentage (%) | numbers | Percentage (%) |
| *Enterococcus* | 125 | 10.40 | 39 | 17.41 |
| *Escherichia* | 123 | 10.23 | 74 | 33.04 |
| *Klebsiella* | 82 | 6.82 | 63 | 28.13 |
| *Staphylococcus* | 71 | 5.91 | 32 | 14.29 |
| *Acinetobacter* | 62 | 5.16 | 29 | 12.95 |
| *Pseudomonas* | 53 | 4.41 | 31 | 13.84 |
| *Clostridium* | 50 | 4.16 | 12 | 5.36 |
| *Psychrobacter* | 37 | 3.08 | 8 | 3.57 |
| *Lactobacillus* | 35 | 2.91 | 11 | 4.91 |
| *Proteus* | 34 | 2.83 | 25 | 11.16 |
| *Bacteroides* | 32 | 2.66 | 10 | 4.46 |
| *Citrobacter* | 28 | 2.33 | 40 | 17.86 |
| *Streptococcus* | 25 | 2.08 | 14 | 6.25 |
| *Enterobacter* | 18 | 1.50 | 21 | 9.38 |
| *Aeromonas* | 16 | 1.33 | 11 | 4.91 |
| *Corynebacterium* | 16 | 1.33 | 10 | 4.46 |
| *Campylobacter* | 15 | 1.25 | 6 | 2.68 |
| *Providencia* | 14 | 1.16 | 10 | 4.46 |
| Clostridioides | 11 | 0.92 | 6 | 2.68 |
| u_Gammaproteobacteria | 10 | 0.83 | 7 | 3.13 |

## Table S5 The host bacteria of top 20 ARG subtypes.

| ARG subtypes | ARCs | | Number of hosts  (genus-level) | Phylum of hosts |
| --- | --- | --- | --- | --- |
|  | Number | Percentage (%) |  |  |
| *bacA*^#^ | 52 | 4.33 | 28 | Firmicutes, Proteobacteria |
| *aadA*^#^ | 42 | 3.49 | 20 | Proteobacteria, Actinobacteria |
| *aadE*^#^ | 39 | 3.24 | 13 | Firmicutes, Proteobacteria |
| *Cat* | 32 | 2.66 | 10 | Proteobacteria, Firmicutes |
| chloramphenicol exporter^#^ | 28 | 2.33 | 13 | Proteobacteria, Actinobacteria, Firmicutes, Bacteroidetes |
| *vanS* | 28 | 2.33 | 1 | Firmicutes |
| *catB* | 27 | 2.25 | 12 | Proteobacteria |
| *tetP* | 27 | 2.25 | 6 | Firmicutes |
| *dfrA1* | 24 | 2.00 | 13 | Proteobacteria |
| *ermG* | 24 | 2.00 | 10 | Firmicutes |
| *vanR*^*^ | 24 | 2.00 | 15 | Actinobacteria, Firmicutes, Proteobacteria |
| *lnuA*^#^ | 22 | 1.83 | 3 | Firmicutes |
| *aac(6')-I* | 20 | 1.66 | 9 | Proteobacteria, Firmicutes |
| cAMP-regulatory protein | 20 | 1.66 | 11 | Proteobacteria |
| *mefA* | 20 | 1.66 | 13 | Firmicutes, Bacteroidetes |
| multidrug_transporter | 20 | 1.66 | 8 | Proteobacteria, Firmicutes |
| *aph(3')-I*^#^ | 19 | 1.58 | 8 | Proteobacteria, Actinobacteria |
| *ermF*^#^ | 19 | 1.58 | 10 | Bacteroidetes |
| *mexT* | 19 | 1.58 | 3 | Proteobacteria |
| *fosB*^*^ | 17 | 1.41 | 7 | Firmicutes, Proteobacteria |

#The average relative abundances of ARGs were in the top 20 list (Figure 3b).

*The relative abundances of ARGs in compost were significantly higher than those in manure samples. (Figure S3)

## Table S6 The number and percentage of MGEs-carried ARCs.

| MGEs | Contigs numbers | Percentage of ARCs |
| --- | --- | --- |
| **Integron** | 102 | 8.50% |
| **Transposase** | 197 | 16.33% |
| **Recombinase** | 37 | 3.08% |
| **Plasmid** | 525 | 43.68% |
| **Total MGEs** | 695 | 57.92% |

## Reference

Bastian, M., S. Heymann and M. Jacomy (2009). Gephi: An Open Source Software for Exploring and Manipulating Networks. Proceedings of the Third International Conference on Weblogs and Social Media, ICWSM 2009, San Jose, California, USA, May 17-20, 2009.

Chen, L., Z. Xiong, L. Sun, J. Yang and Q. Jin (2011). "VFDB 2012 update: toward the genetic diversity and molecular evolution of bacterial virulence factors." Nucleic Acids Research **40**(D1): D641-D645.

Cury, J., T. Jové, M. Touchon, B. Néron and E. P. Rocha (2016). "Identification and analysis of integrons and cassette arrays in bacterial genomes." Nucleic acids research **44**(10): 4539-4550.

Forsberg, K. J., S. Patel, M. K. Gibson, C. L. Lauber, R. Knight, N. Fierer and G. Dantas (2014). "Bacterial phylogeny structures soil resistomes across habitats." Nature **509**: 612.

Fresia, P., V. Antelo, C. Salazar, M. Giménez, B. D’Alessandro, E. Afshinnekoo, C. Mason, G. H. Gonnet and G. Iraola (2019). "Urban metagenomics uncover antibiotic resistance reservoirs in coastal beach and sewage waters." Microbiome **7**(1): 35.

Fu, L., B. Niu, Z. Zhu, S. Wu and W. Li (2012). "CD-HIT: accelerated for clustering the next-generation sequencing data." Bioinformatics **28**(23): 3150-3152.

Li, C., Y. Li, X. Li, X. Ma, S. Ru, T. Qiu and A. Lu (2020). "Veterinary antibiotics and estrogen hormones in manures from concentrated animal feedlots and their potential ecological risks." Environmental Research: 110463.

Li, D., C. M. Liu, R. Luo, K. Sadakane and T. W. Lam (2015). "MEGAHIT: an ultra-fast single-node solution for large and complex metagenomics assembly via succinct de Bruijn graph." Bioinformatics **31**(10): 1674-1676.

Lu, J., F. P. Breitwieser, P. Thielen and S. L. Salzberg (2016). "Bracken: Estimating species abundance in metagenomics data." bioRxiv: 051813.

Ma, L., Y. Xia, B. Li, Y. Yang, L.-G. Li, J. M. Tiedje and T. Zhang (2016). "Metagenomic Assembly Reveals Hosts of Antibiotic Resistance Genes and the Shared Resistome in Pig, Chicken, and Human Feces." Environmental science & technology **50**(1): 420-427.

Patro, R., G. Duggal, M. I. Love, R. A. Irizarry and C. Kingsford (2017). "Salmon provides fast and bias-aware quantification of transcript expression." Nat Methods **14**(4): 417-419.

Seemann, T. (2014). "Prokka: rapid prokaryotic genome annotation." Bioinformatics **30**(14): 2068-2069.

Uritskiy, G. V., J. DiRuggiero and J. Taylor (2018). "MetaWRAP—a flexible pipeline for genome-resolved metagenomic data analysis." Microbiome **6**(1): 158.

Wood, D. E. and S. L. Salzberg (2014). "Kraken: ultrafast metagenomic sequence classification using exact alignments." Genome Biology **15**(3): R46.

Yang, Y., X. Jiang, B. Chai, L. Ma, B. Li, A. Zhang, J. R. Cole, J. M. Tiedje and T. Zhang (2016). "ARGs-OAP: online analysis pipeline for antibiotic resistance genes detection from metagenomic data using an integrated structured ARG-database." Bioinformatics **32**(15): 2346-2351.

Yin, X., X.-T. Jiang, B. Chai, L. Li, Y. Yang, J. R. Cole, J. M. Tiedje and T. Zhang (2018). "ARGs-OAP v2. 0 with an expanded SARG database and Hidden Markov Models for enhancement characterization and quantification of antibiotic resistance genes in environmental metagenomes." Bioinformatics **1**: 8.
